# Survival of intracellular pathogens in response to mTORC1- or TRPML1-TFEB-induced xenophagy

**DOI:** 10.1080/27694127.2023.2191918

**Published:** 2023-03-19

**Authors:** Mariana I. Capurro, Akriti Prashar, Xiaodong Gao, Nicola L. Jones

**Affiliations:** a Program in Cell Biology, Peter Gilgan Centre for Research and Learning, The Hospital for Sick Children, Toronto, Ontario, Canada; b Division of Gastroenterology, Hepatology and Nutrition, The Hospital for Sick Children, Toronto, Ontario, Canada; c Departments of Paediatrics and Physiology, University of Toronto, Toronto, Ontario, Canada

**Keywords:** Autophagy, infection, invasive bacteria, ML-SA1, mTORC1, rapamycin, TFEB, TRPML1

## Abstract

Intracellular pathogens establish persistent infections by generating reservoirs that protect them from the action of antibiotics and the host immune response. Novel therapeutics should then target the host pathways exploited by the pathogens to form these intracellular niches. An attractive strategy to achieve this is inducing xenophagy, the selective autophagy that recognizes and targets invading pathogens for degradation. However, some bacteria have evolved mechanisms to co-opt xenophagy for their own benefit. Therefore, in this study we determine the effect of inducing xenophagy by different pathways, namely the inhibition of MTOR or through TRPML1-TFEB activation, on the fate of pathogens that are either susceptible to, evade or require autophagy for intracellular survival. We identified a dose of rapamycin that exclusively induces autophagy through MTOR inhibition and used ML-SA1 to activate the TRPML1-TFEB pathway, which also increases lysosomal biogenesis. We found that ML-SA1 induced greater autophagy flux than rapamycin. By performing in vitro infections with *H. pylori, S*. Typhimurium, *S. flexneri, L. monocytogenes* and *S. aureus*, we established that ML-SA1 had a more potent effect than rapamycin in restricting the growth of pathogens susceptible to xenophagy. In the case of pathogens that produce effectors to block xenophagy, ML-SA1, but not rapamycin, resulted in bacterial killing. During *S. aureus* infection, which depends on autophagy for intracellular survival, ML-SA1 administration potentiated bacterial growth. We suggest that while targeting the xenophagy pathway holds promise for treatment of intracellular pathogens, a precision approach to select the correct target to induce effective bacterial killing is warranted.

**Abbreviations**: 3-MA: 3-methyladenine, ATG: autophagy-related protein, Baf: bafilomycin A1; Ca2+: calcium, CFU: colony-forming units, DMSO: dimethyl sulfoxide, h: hour, *Hp: Helicobacter pylori*, hpi: hours post-infection, Lamp1: lysosomal-associated membrane protein 1, LC3: microtubule-associated protein 1A/1B-light chain, *Lm: Listeria monocytogenes*, LSD: lysosomal storage disorder, min: minutes, mTOR: mechanistic target of rapamycin; mTORC1: mechanistic target of rapamycin complex 1, MEF: mouse embryonic fibroblast, μM: micromolar, moi: multiplicity of infection, nM: nanomolar, OD: optical density, PBS: phosphate buffer saline, *Sa: Staphylococcus aureus*, SCV: *Salmonella* containing vacuole, Sifs: *Salmonella*-induced filaments, *Sf: Shigella flexneri*, SLAPs: Spacious Listeria containing phagosomes, *St: Salmonella* Typhimurium TFEB: transcription factor EB, TRPML1: transient receptor potential membrane channel 1, VacA: vacuolating cytotoxin, wt: wild-type.

## Introduction

Infectious diseases pose a major threat to human health ^1^. Of special concern for treatment failure is the rise of antibiotic-resistant bacteria ^2^. In addition, some susceptible pathogens establish persistent infections due to their ability to invade host cells and generate intracellular reservoirs that protect them from the action of antibiotics and the immune response. Therefore, alternative approaches to target pathogens while preventing antibiotic resistance are needed. There is currently great interest in host-directed therapeutics that target the specific host pathways that are exploited by a pathogen to mediate their intracellular survival. These host-directed therapeutics are expected to overcome antibacterial resistance ^3,4^.

Invasive bacteria enter the host cell via phagocytosis, endocytosis or micropinocytosis and encounter two main pathways involved in degradation of intracellular pathogens, the lysosomal and the autophagy pathways ^5^. A variety of pathogens have evolved mechanisms to avoid lysosomal killing such as escaping the lysosome, blocking fusion of the bacteria-containing compartment with lysosomes or altering the degradative capacity of lysosomes ^6,7^. The presence of intracellular bacteria can also trigger the cell autophagic response. Autophagy is initiated by the formation and expansion of a membrane enclosing the cargo, called phagophore; followed by the fusion of its edges to make the autophagosome. The autophagosome then fuses with lysosomes to form the autolysosome where the captured material, along with the inner membrane, is degraded ^8^. The selective autophagy that recognizes and targets invading pathogens is named xenophagy ^9^, and is considered a part of the innate immune response ^9–11^. Thus, xenophagy induction has been proposed as an attractive strategy to eliminate intracellular pathogens ^10,11^. However, several pathogens have evolved mechanisms to evade or block the xenophagic response. Some pathogens even actively trigger xenophagy and hijack its components to promote their survival ^10,90,12–15^. Thus, understanding the complex pathogen-host interaction is needed to effectively target and kill intracellular pathogens.

The most common way to modulate autophagy is via MTOR (mechanistic target of rapamycin). The kinase complex mTORC1 represses autophagy through the phosphorylation of multiple autophagy-related proteins (ATG) involved in phagophore initiation, elongation and autophagosome formation ^16–18^. Therefore, pharmacologic inhibition of mTORC1 triggers autophagy by inducing autophagosome formation. However, overcoming persistent infections in vivo needs a sustained autophagy-mediated killing of intracellular bacteria, requiring concomitant lysosomal activation, reformation and biogenesis to process the increase in autophagosome formation ^19–21^. Consequently, the induction of autophagy by mTORC1 inhibition might not be a sufficient strategy. Similarly, in the case of pathogens that impair lysosomal function, autophagy induction alone is unlikely to induce a sustained autophagic flux and bacterial clearance ^6,7,22^.

Studies performed in several lysosomal storage disorder (LSD) and neurodegenerative disease models suggest that enhancing both lysosomal and autophagic functions by overexpression/activation of Transcription Factor EB (TFEB) could be a more beneficial therapeutic approach ^21,23–25^. TFEB is the master regulator of the lysosome to nucleus signaling pathway that jointly coordinates autophagy and lysosomal gene expression ^21,26,27^. TFEB phosphorylation status controls its subcellular localization and activity. While phosphorylated (inactive) TFEB is mainly cytoplasmic, it rapidly translocates to the nucleus upon de-phosphorylation by the phosphatase calcineurin ^28,29^. In response to lysosomal perturbations, the endolysosomal calcium (Ca2+) channel TRPML1 (transient receptor potential membrane channel 1) mediates the transient, localized Ca2+ release that leads to calcineurin activation and TFEB dephosphorylation ^29^. TRPML1 activity is essential for maintaining lysosomal ion homeostasis and membrane trafficking events between lysosomes and autophagosomes, late endosomes and plasma membrane ^30,31^. Thus, TRPML1 activation enhances lysosomal function and calcineurin-mediated TFEB activation resulting in induction of TFEB target genes and autophagy flux enhancement.

Rapamycin is the most commonly used drug to experimentally induce autophagy due to its inhibitory effect on mTORC1. Rapamycin specifically binds to mTORC1 with high affinity (nanomolar (nM) range) and allosterically inhibits its kinase activity ^22,32,33^. Although rapamycin has been used in several in vitro models of infection with promising effects on intracellular bacterial clearance ^34–38^, it was recently reported that rapamycin at micromolar (μM) concentrations directly binds and activates TRPML1 ^22^. As most studies on xenophagy are performed with μM doses of rapamycin ^34–38^, a reinterpretation of these results is needed since the beneficial effect of rapamycin attributed to mTOR pathway may indeed be TRPML1-TFEB-mediated. Furthermore, rapamycin is also used for immunosuppression to prevent solid organ rejection^39^ and may have unwanted effects during infection. Therefore, if the effects of rapamycin on pathogen clearance were indeed a consequence of TRPML1 activation, a TRPML1 agonist with no effect on mTORC1 activity would be a more suitable drug to treat infections.

Here, we assessed the effect of inducing xenophagy in a mTORC1-dependent vs. TRPML1-TFEB-mediated manner on the fate of pathogens that are susceptible to, evade or require autophagy for intracellular survival.

## Results

### Micromolar dose of rapamycin and TRPML1 agonist ML-SA1 activate TRPML1-TFEB axis

To dissect the effect of xenophagy mediated through either the mTORC1 or TRPML1-TFEB axes, we first identified exclusive inducers of each pathway. In general, mTORC1 is inhibited by rapamycin and TRPML1 activated by small molecules agonists like ML-SA1 ^40^. However, based on the recently reported effect of high dose rapamycin on TRPML1 ^22^, we first established a rapamycin concentration that inhibited mTORC1 with no effect on TRPML1, and confirmed that the TRPML1 agonist ML-SA1 does not modify mTORC1 activity.

**Figure 1.**
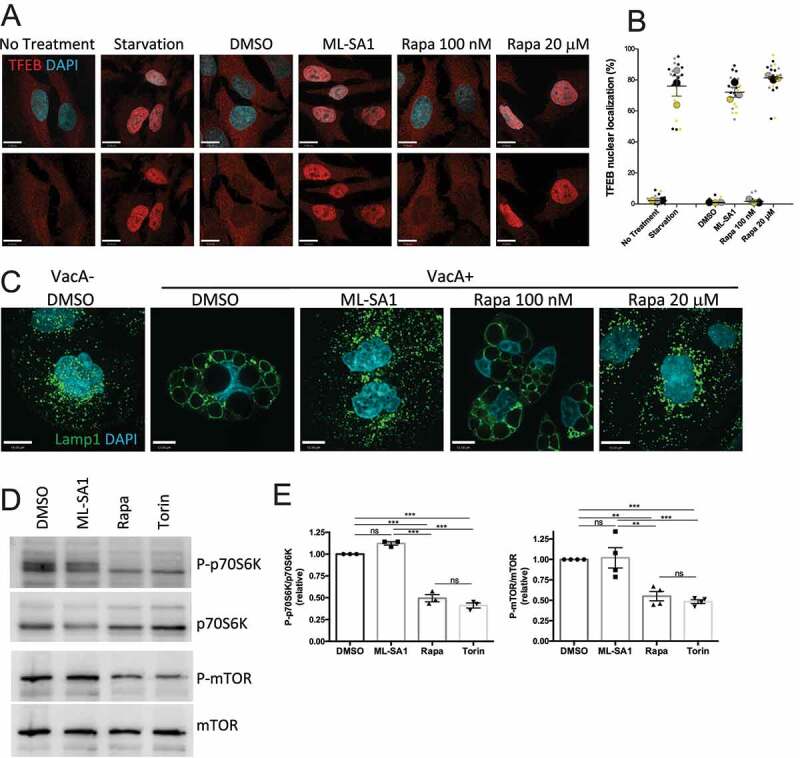
Micromolar dose of rapamycin and TRPML1 agonist ML-SA1 activate TRPML1-TFEB axis. (**A**) TFEB staining of HeLa cells after 2 h starvation or treatment with ML-SA1 (20 μM), rapamycin (Rapa) (100 nM or 20 μM) or vehicle control (DMSO). (**B**) Quantification of cells from A, expressed as percentage of cells with nuclear TFEB (n=3); each color corresponds to 1 experiment, big symbol represent the mean and small symbols the scatter dot plot. Lines show the mean +/- SEM. (**C**) Lamp1 staining of AGS cells after 4h VacA- or VacA+ incubation followed by 3h of DMSO, ML-SA1 or rapamycin (Rapa) treatment. (**D**) Western blotting for p70S6K and MTOR phosphorylation after 3h treatment with DMSO, ML-SA1 (20 μM), rapamycin (Rapa, 100 nM) or Torin (1 μM). (**E**) Graphs show quantification of phospho-proteins normalized to total protein levels (mean +/- SEM of 3 (for p70S6K) or 4 (for mTOR) different experiments).

We compared the effect of rapamycin at high (20 μM) and low (100 nM) concentrations on TFEB activation by assessing its nuclear translocation using immunofluorescence staining. We found that rapamycin at μM concentration induced a strong nuclear translocation of TFEB comparable to the effects of the TRPML1 agonist ML-SA1 and starvation, both known activators of TFEB. Of note, mTORC1-inhibiting nM concentrations of rapamycin did not induce TFEB nuclear translocation (Figure 1A,B).

Next, in complementary studies we investigated the effect of the two different concentrations of rapamycin using a direct readout of TRPML1 activation. We previously established that the vacuolating toxin (VacA) secreted by *Helicobacter pylori* (*Hp*) impairs TRPML1 activity and induces cell vacuolation in AGS (gastric adenocarcinoma) cells, as assessed by Lamp1 (lysosomal-associated membrane protein 1) immunolabelling (Figure 1C) ^41–43^. TRPML1 activation by ML-SA1 rapidly restores vesicular trafficking and reverses vacuolation leading to the formation of functional lysosomes (Figure 1C) ^41–43^. In addition, ML-SA1 leads to TFEB activation in AGS cells as assessed by its nuclear translocation and phosphorylation status (Figure S1). We found that administration of rapamycin at high (μM), but not low (nM) concentrations reversed VacA vacuolation, confirming that rapamycin at high dose activates TRPML1 (Figure 1C). As a control, Torin, a strong catalytic inhibitor of mTORC1 that does not interact with TRPML1^22^ has no effect on VacA-induced vacuolation (Figure S2A). Finally, we verified that ML-SA1 does not affect mTORC1 activity by assessing the relative levels of MTOR and p70S6K phosphorylation, commonly used markers for monitoring mTORC1 kinase activity^44^ following ML-SA1 treatment (Figure 1D,E). As controls, both 100 nM rapamycin and Torin displayed an mTORC1 inhibitory effect (Figure 1D,E). Altogether, we established that 100 nM rapamycin inhibits mTORC1 without activating the TRPML1-TFEB axis whereas 20 μM ML-SA1 activates TRPML1 with no effect on mTORC1.

### TRPML1-TFEB activation, but not MTOR inhibition, increases autophagy flux

**Figure 2.**
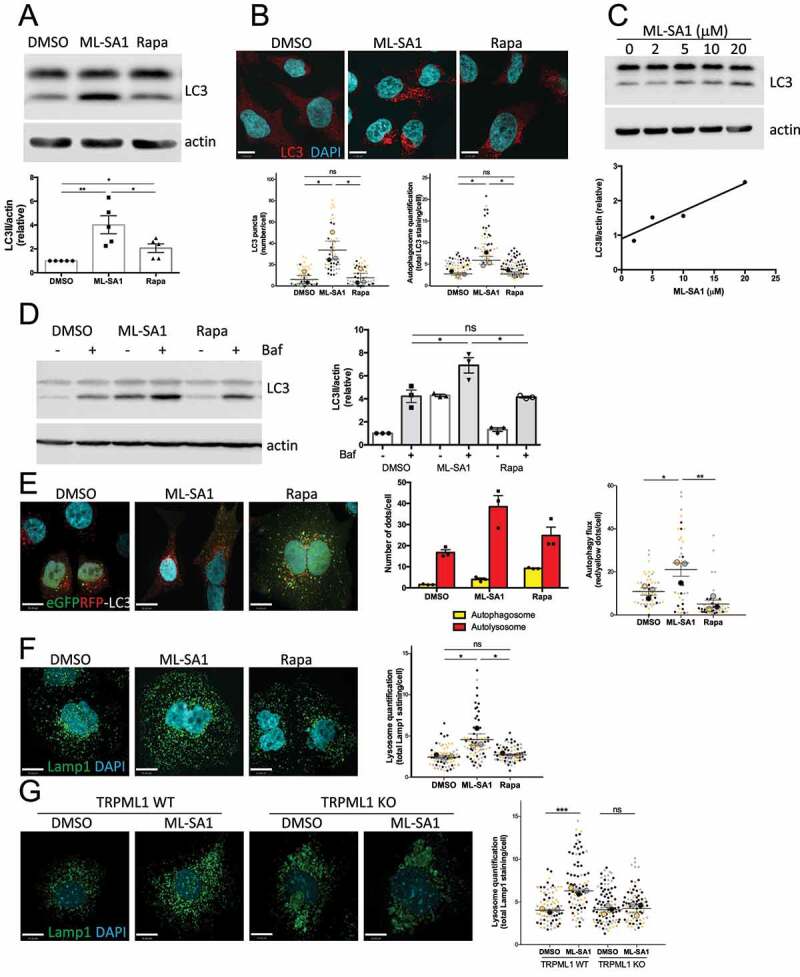
TRPML1-TFEB activation, but not MTOR inhibition, increases autophagy flux. (**A**) LC3 western blotting of HeLa cells treated with DMSO, ML-SA1 (20 μM) or rapamycin (Rapa, 100 nM) for 4h using actin as loading control. The graph shows quantification of LC3II normalized to actin (mean +/- SEM of 5 different experiments). (**B**) LC3 staining of cells treated as in (A). Graphs show the autophagosome quantification indicating the number of LC3 puncta/cell (left) and total LC3 staining/cell (right), (n=3; each color corresponds to 1 experiment, big symbol representing the mean and small symbols the scatter dot plot. Lines show the mean +/- SEM). (**C**) LC3 western blotting of HeLa cells treated with different concentrations of ML-SA1 for 4h using actin as loading control. Graph shows the dose-dependent LC3II induction. (**D**) LC3 western blotting of cells after 4h treatment with DMSO, ML-SA1 (20 μM) or rapamycin (Rapa, 100 nM), followed with 2 h Bafilomycin A1 (Baf, 100 nM) incubation. The graph shows quantification of LC3II normalized to actin (mean +/- SEM of 3 different experiments). (**E**) Images of HeLa cells expressing the tandem eGFPRFP-LC3 reporter treated as in (B). Yellow puncta correspond to autophagosomes (AP), red puncta correspond to autolysosomes (AL). Graphs show the number of AP and AL/cell and AL/AP ratio. (**F**) Lamp1 staining of cells treated as in (B). Graph shows lysosome quantification (total Lamp1 staining/cell, n=3; each color corresponds to 1 experiment, big symbol representing the mean and small symbols the scatter dot plot. Lines show the mean +/- SEM). (**G**) Lamp1 staining of TRPML1 KO and wild-type MEFs treated with DMSO and ML-SA1 (20 μM). Lysosome quantification performed as is (**F**).

We next compared the effect of rapamycin-mTORC1 and ML-SA1-TRPML1 pathways on autophagy induction by measuring the levels of the phagophore and autophagosomal membrane-associated protein LC3II (microtubule-associated protein 1A/1B-light chain) by immunoblotting and immunofluorescence staining. We found that ML-SA1 administration to HeLa cells produced a stronger effect on autophagy induction than nM levels of rapamycin (Figure 2A,B and S3). ML-SA1 displayed a dose-dependent effect with greater LC3II accumulation detected at 20 μM (Figure 2C), the concentration chosen for further experiments. As LC3II accumulation may also occur due to a block in autophagosome degradation, to properly assess autophagic flux HeLa cells treated with ML-SA1 or rapamycin (100 nM) were then exposed to the vacuolar H^+^-ATPase inhibitor bafilomycin A1 (Baf), which blocks lysosomal function ^45^. Baf administration after ML-SA1, but not rapamycin (100 nM) treatment, produced an increase in LC3II levels as compared to Baf alone (Figure 2D), confirming that TRPML1-TFEB activation by ML-SA1 indeed increased autophagy flux. In contrast, mTORC1 inhibition by rapamycin (100 nM) was sufficient to induce autophagy but not to increase autophagy flux (Figure 2A,D). To complement these findings, we compared the effect of ML-SA1 and rapamycin (100 nM) treatments in HeLa cells transfected with a GFP-RFP-LC3 tandem construct. As GFP loses fluorescence in the acidic lysosomal environment, but RFP does not, the GFP-RFP LC3 tandem is commonly used to study the maturation of autophagosomes (GFP and RFP signal) into autolysosomes (RFP signal only) ^46^. We found that administration of ML-SA1 to cells produced an increased number of autolysosomes vs. autophagosomes in comparison with cells treated with rapamycin (100 nM) (Figure 2E). In agreement with this observation, Lamp1 staining revealed that ML-SA1, but not rapamycin (100 nM), triggered lysosomal biogenesis (Figure 2F). Studies performed in TRPML1 knockout (KO) murine embryonic fibroblasts (MEFs) confirmed that the effect of ML-SA1 on lysosomal numbers is TRPML1-dependent, as ML-SA1 treatment did not result in an increase in lysosomes in TRPML1 knockout cells (Figure 2G). Collectively, we showed that activation of the TRPML1-TFEB axis by ML-SA1 increased autophagy flux as compared with rapamycin (100 nM)-mediated mTORC1 inhibition.

### Differential effect of autophagy induction by TRPML1 activation or mTORC1 inhibition on Hp intracellular survival

*Hp* growth is restricted by xenophagy ^47,48^. Autophagy-deficient Atg5 KO (Atg5-/-) MEFs are more permissive for *Hp* intracellular survival than the wild-type (wt) counterparts, and individuals with *Atg16L1* mutation show an increased susceptibility to *Hp* infections ^48^. However, the *Hp* virulence factor VacA inhibits TRPML1, thereby, disrupting lysosomal trafficking and autophagosome maturation to generate an intracellular niche for *Hp* ^41,43,48,49^. Therefore, *Hp* infection represents an ideal model to compare, as proof of principle, the differential effect of TRPML1- and mTORC1-mediated xenophagy on intracellular bacterial clearance. We have previously reported that TRPML1 activation by ML-SA1 in *Hp*-infected AGS cells reverses the effects of VacA resulting in restoration of endolysosomal and xenophagy pathways, leading to the efficient killing of intracellular *Hp* ^43^. Furthermore, we confirmed previously that ML-SA1 effect on bacterial killing is mediated by TRPML1, as ML-SA1 had no effect on bacterial eradication when administered to *Hp*-infected gastric organoids obtained from TRPML1 KO mice ^43^. Therefore, we performed gentamycin protection assays to compare the intracellular survival of *Hp* in infected AGS cells treated with ML-SA1 or doses of rapamycin that either inactivated mTORC1 (100nM) or activated TRPML1 (μM) using colony forming units (CFU).

**Figure 3.**
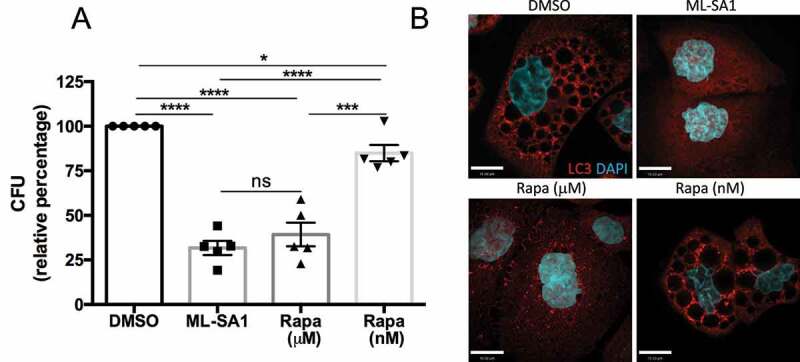
Differential effect of autophagy induction by TRPML1 activation or mTORC1 inhibition on *Hp* intracellular survival. (**A**) AGS cells infected with wild-type (VacA+) *Hp* were incubated with gentamycin to kill extracellular bacteria and treated with ML-SA1 (20 μM), rapamycin (Rapa, 20 μM [μM] or 100 nM [nM]) or vehicle control (DMSO). Intracellular bacteria were retrieved and CFUs quantified. Graph shows relative percentage of CFU (mean +/- SEM of 5 independent experiments) considering 100 the bacteria retrieved from DMSO-treated cells. (**B**) LC3 staining of AGS cells after 4h VacA+ incubation followed by 3h of DMSO, ML-SA1 or rapamycin treatment.

Although both doses of rapamycin significantly reduced CFU counts in *Hp* infected cells, the effect of rapamycin at μM concentration was more pronounced, and comparable to the reduction in CFU caused by ML-SA1 administration (Figure 3A). LC3 and Lamp1 staining of cells exposed to VacA toxin and treated under the different conditions confirmed that rapamycin (μM) restores vesicular trafficking (Figure 1C) and autophagy flux (Figure 3B) at levels comparable to treatment with ML-SA1 (Figure 1C, and 3B). Importantly, we confirmed that the effect of ML-SA1 on intracellular bacterial killing was not due to a direct antimicrobial action since *Hp* was able to grow in the presence of the compound (Figure S4). Altogether, these data indicate that similar to ML-SA1, the more potent effect of rapamycin at μM levels on intracellular bacterial killing is mediated by TRPML1 activation. By contrast, restricting the effect of rapamycin to mTORC1 inhibition, by using nM concentrations, leads only to a limited reduction of *Hp* growth. Consistent with these observations, inducing xenophagy by the mTORC1 catalytic inhibitor Torin, at doses that inhibited mTOR to a similar extent as μM Rapamycin (Figure S2B), produced a limited decrease in CFU counts that was comparable to rapamycin treatment at the nM level (Figure S2C). As expected, ML-SA1-mediated *Hp* eradication correlated with reduced levels of CagA, the main *Hp* virulence factor associated with carcinogenesis, in the cell lysate. (Figure S2D).

### Effect of MTOR- or TRPML1- dependent pathways on bacteria susceptible to xenophagy

We next investigated the effect of ML-SA1 and mTORC1-inhibiting rapamycin dose (100 nM) on the fate of *Salmonella* Typhimurium (*St*) and *Listeria monocytogenes* (*Lm*), two pathogens that are restricted by xenophagy during the early phase of their intracellular lifestyle, but escape xenophagy at later time points. *St* is transiently susceptible to autophagy, but then hijacks the mTORC1 complex to evade xenophagy. Early after infection (1h post-infection [hpi]), *St* that has invaded epithelial cells resides in *Salmonella*-containing vacuoles (SCV). A proportion of SCVs are damaged via the type III secretion system (T3SS) allowing the bacteria to access cytosolic nutrients, resulting in acute amino acid starvation, mTORC1 inhibition and induction of autophagy that targets the damaged vacuole and bacteria within ^50,12^. By 4 hpi, the cytosolic amino acid pool is restored and mTORC1 is reactivated. *St* then promotes the recruitment of the mTORC1 complex to the SCV membrane, thus inhibiting xenophagy ^36,51^ and replicating in the SCV ^52^. At later stages of infection (> 8 hpi) tubular membranous extensions, called *Salmonella*-induced filaments (Sifs), protrude from the SCV.

**Figure 4.**
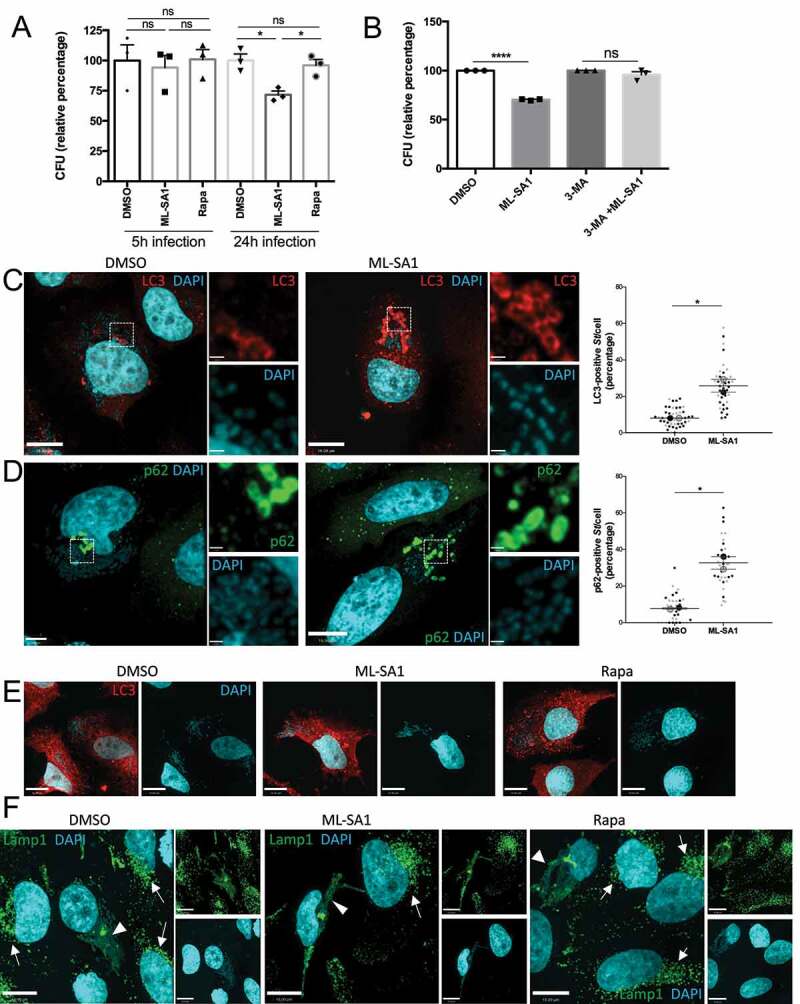
Effect of MTOR and TRPML1 pathways on *St* intracellular survival. (**A**) HeLa cells infected with *St* were treated for 5h or 24 h with ML-SA1 (20 μM), rapamycin (Rapa, 100 nM) or vehicle control (DMSO). Intracellular bacteria were retrieved and CFU quantified. Graph shows relative percentage of CFU (mean +/- SEM of 3 independent experiments) considering 100 the bacteria retrieved from DMSO-treated cells. (**B**) HeLa cells infected with *St* were treated with DMSO, ML-SA1 (20 μM), 3-methyladenine (3-MA) or 3-MA containing 20 μM ML-SA1 (3-MA + ML-SA1). Intracellular bacteria were retrieved and CFU quantified as in (**A**). (**C-D**) LC3 (**C**) or p62 (**D**) staining of HeLa cells infected with *St* and treated with DMSO or ML-SA1 (20 μM) for 4h. DAPI staining used to visualize bacteria. Higher magnifications of the selected area in the separate channels are included to the right. Graphs show the percentage of bacteria positive for each marker. Each color corresponds to 1 experiment, big symbols represent the mean and the small symbols the scatter dot plot/experiment. Lines show the mean +/- SEM. (**E**) LC3 staining of *St*-infected cells treated with DMSO or ML-SA1 (20 μM) for 24h. Images of DAPI channel to visualize bacteria are included at the right. (**F**) Lamp1 staining of cells infected and treated as in (E). Arrows point to non-infected cells, arrowheads to SCV and Sifs. Images from the separate channels included at the right.

Enhancing TRPML1-dependent xenophagy by ML-SA1 in *St*-infected HeLa cells did not affect bacterial survival during short-term infections (5 h), but significantly limited intracellular *St* growth in long-term (24 h) infections (Figure 4A). In contrast, inhibition of mTORC1 by rapamycin (100 nM) did not affect *St* growth at either time point (Figure 4A). We confirmed that ML-SA1 effect on *St* growth was dependent on autophagy activation, as ML-SA1 failed to alter *St* survival in the presence of the autophagy inhibitor 3-methyladenine (3-MA) (Figure 4B). Furthermore, ML-SA1 increased the recruitment of LC3 to intracellular bacteria (Figure 4C). Efficient xenophagy of *St* requires the autophagy adaptor SQSTM1/p62 (p62) that binds ubiquitinated bacteria via its ubiquitin associated domain (UBA) and LC3 through a LC3 interacting region (LIR) ^53^. Phosphorylation of S403 in the UBA domain of p62 by TBK1 (TANK-binding kinase 1) increases the binding of p62 to ubiquitinated cargoes. Activation of TBK1 involves its phosphorylation at S172 ^54^. Therefore, we determined the recruitment of p62, S403-P-p62 and S172-P-TBK1 to *St* to further assess the capture of ubiquitinated *St* by autophagosomes. As shown in Figure 4D and S5A,B, ML-SA1 administration significantly increased the amount of p62-, P-p62- and P-TBK1-positive *St*. Altogether, these results indicate that TRPML1 activation by ML-SA1 increased the cellular xenophagic response to *St* infection. We then performed LC3 and Lamp1 immunostaining to determine the identity of the bacterial-containing compartment at 24 hpi. Of note, most of the intracellular *St* in control (DMSO-treated) infected cells were not captured in LC3 positive autophagosomes at this time point (Figure 4E), but instead were present within Lamp1-positive SCV/Sifs (Figure 4F). Notably, in comparison with non-infected neighbouring cells, *St*-infected cells had markedly reduced number of lysosomes and instead Lamp1-positive membranes were located on the SCVs/Sifs (Figure 4F). These results suggest that the beneficial effect of ML-SA1 may be due to the TRPML1-TFEB-mediated increase in lysosomal population, leading to a reduction in the number and/or size of SCVs/Sifs. The lack of effect of rapamycin on *St* clearance may reflect its inability to activate TRPML1-TFEB axis at nM concentration. In agreement with this possibility, when administered at TRPML1-activating μM doses, rapamycin was as effective as ML-SA1 in reducing intracellular bacterial survival (Figure S5C).

*Lm* is another prototypical pathogen that is transiently susceptible to autophagy. *Lm* infection induces an early xenophagic response that inhibits the growth of the bacteria, as demonstrated by the 4 h growth delay reported in Atg5 wt compared to Atg5-/- MEFs ^55^. However, at later time points, *Lm* is able to evade xenophagy ^56^. *Lm* escapes from the phagosome by using the pore-forming toxin listeriolysin O (LLO), and once in the cytosol the effector ActA recruits host actin to the bacterial surface masking *Lm* from xenophagy recognition and allowing rapid bacterial replication ^13,56^. *Lm* that express low LLO levels fail to sufficiently damage the phagosome or escape into the cytosol and LC3 is recruited to these damaged phagosomes. However, the small LLO-generated pores uncouple the pH gradient preventing acidification of the compartment, thereby preventing maturation and bacterial degradation ^56^. As a consequence, a percentage of *Lm* is found in large Lamp1+, LC3+ compartments named Spacious Listeria containing phagosomes (SLAPs), which allow slow bacterial replication and persistent infection ^56^.

**Figure 5.**
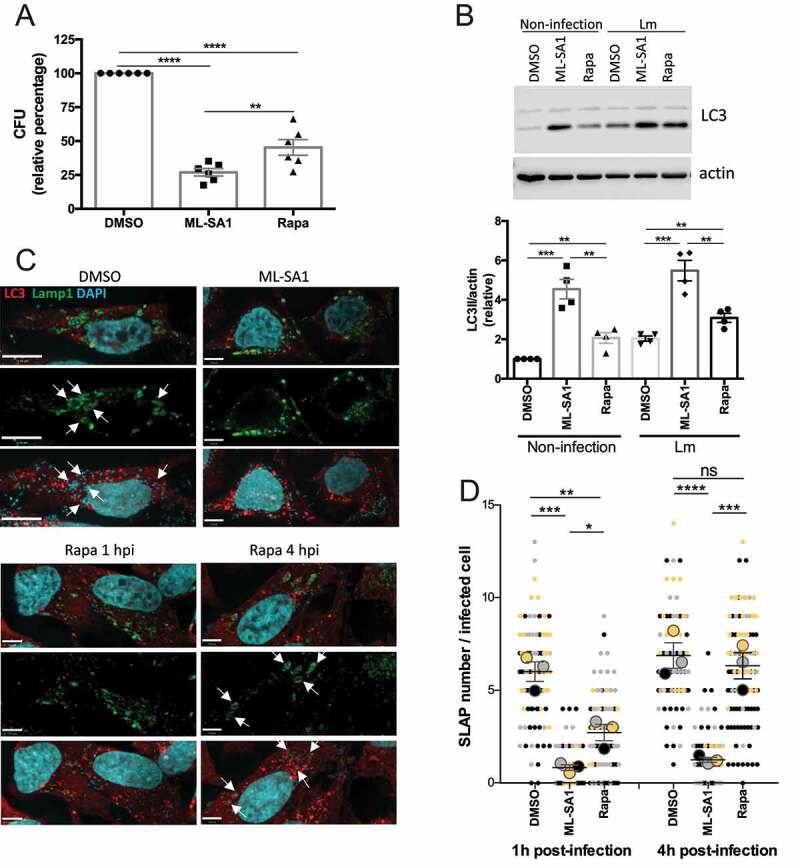
Effect of MTOR or TRPML1-dependent pathways on *Lm* intracellular survival. (**A**) HeLa cells infected with *Lm* were incubated with gentamycin and treated for 23 h with ML-SA1 (20 μM), rapamycin (Rapa, 100 nM) or vehicle control (DMSO). Intracellular bacteria were retrieved and CFUs quantified. Graph shows relative percentage of CFU (mean +/- SEM of 6 independent experiments) considering 100 the bacteria retrieved from DMSO-treated cells. (**B**) LC3 western blotting of *Lm*-infected (Lm) and non-infected control cells (Non-infection) treated as in (A) using actin as loading control. Graph shows quantification of LC3II normalized to actin (mean +/- SEM of 4 different experiments). (**C**) LC3 and Lamp1 staining of cells infected and treated as in (A). For rapamycin-treated cells, panels with drug added 1 h (Rapa 1 hpi) or 4 h (Rapa 4 hpi) after infection are included. Arrows indicate SLAPs. (**D**) SLAP quantification in *Lm*-infected HeLa cells and treated as (C), with drugs added 1h and 4h post-infection (n=3, each color corresponds to 1 experiment, big symbol represent the mean and small symbols the scatter dot plot. Lines show the mean +/- SEM).

We first assessed the effect of triggering xenophagy in *Lm*-infected HeLa cells at early time points (1 hpi). Both TRPML1 activation and mTORC1 inhibition significantly reduced intracellular bacterial survival as quantified by CFU assay (Figure 5A). However, treatment with ML-SA1 reduced bacterial survival to a greater extent than rapamycin (100 nM), which is consistent with the observation that ML-SA1 is a more potent autophagy inducer as assessed by increased LC3 (Figure 5B). The effect of ML-SA1 on *Lm* growth was significantly reduced in the presence of 3-MA, indicating its dependence on autophagy activation (Figure S6A). In agreement with the reduction in bacterial survival, Lamp1 and LC3 immunostaining revealed a decrease in SLAP formation (Figure 5C,D). In comparison to the control (DMSO-treated) group where several SLAPs per cell were detected (Figure 5C, arrows), ML-SA1 treatment reduced SLAP formation to a greater extent (Figure 5C,D) than rapamycin (100 nM) administration. We next assessed whether xenophagy induction plays a beneficial role in bacterial killing at later time points when SLAP formation was more significant, by administering the treatments 4 hpi. Figures 5C,D and S6B show that ML-SA1, but not rapamycin (100 nM), significantly reduced the number of SLAPs at this time point. In rapamycin-treated cells the number of SLAPs were similar to DMSO-treated control cells, but the SLAPs were smaller in size and contained a lower number of *Lm* per SLAP (Figure 5C). Consistent with this observation, a small decrease in CFU was detected when rapamycin (100 nM) was added 4 hpi but was not as marked as the reduction in CFU caused by ML-SA1 treatment (Figure S6C). Altogether these results indicate that mTORC1-induced xenophagy can target *Lm* during the early phase of infection, however, TRPML1 activation is more effective at targeting *Lm* both during early and later stages of infection when SLAPs are more prominent.

Next, we determined whether TRPML1 activation by ML-SA1 increased the cellular xenophagic response by enhancing p62 recognition of ubiquitinated *Lm*. Figure S7 shows that ML-SA1 administration increased the number of LC3-positive *Lm*, without modifying the recruitment of p62, P-p62 or P-TBK1 to intracellular *Lm* indicating an alternate mechanism for enhancing LC3 recruitment.

### Effect of MTOR or TRPML1 pathways on bacteria that depend on autophagy for intracellular survival

*Staphylococcus aureus* (*Sa*) requires autophagy to generate its intracellular replicative niche. The current model contends that *Sa* secretes the pore-forming toxin alpha-hemolysin (Hla) that promotes xenophagy, but after *Sa* is engulfed by an autophagosome, Hla prevents autophagosome maturation and fusion with lysosomes ^9,57^. Thus, *Sa* replicates in a double membrane LC3-positive autophagosome for 3 to 12 hpi. Following replication, the bacteria escape into the cytosol and induce apoptosis of host cells ^13^. In agreement with this model, *Sa* cannot replicate in Atg5-/- MEFs. Similarly, Hla-mutant bacteria, unable to trigger xenophagy, are delivered to and degraded within lysosomes ^12,57^.

**Figure 6.**
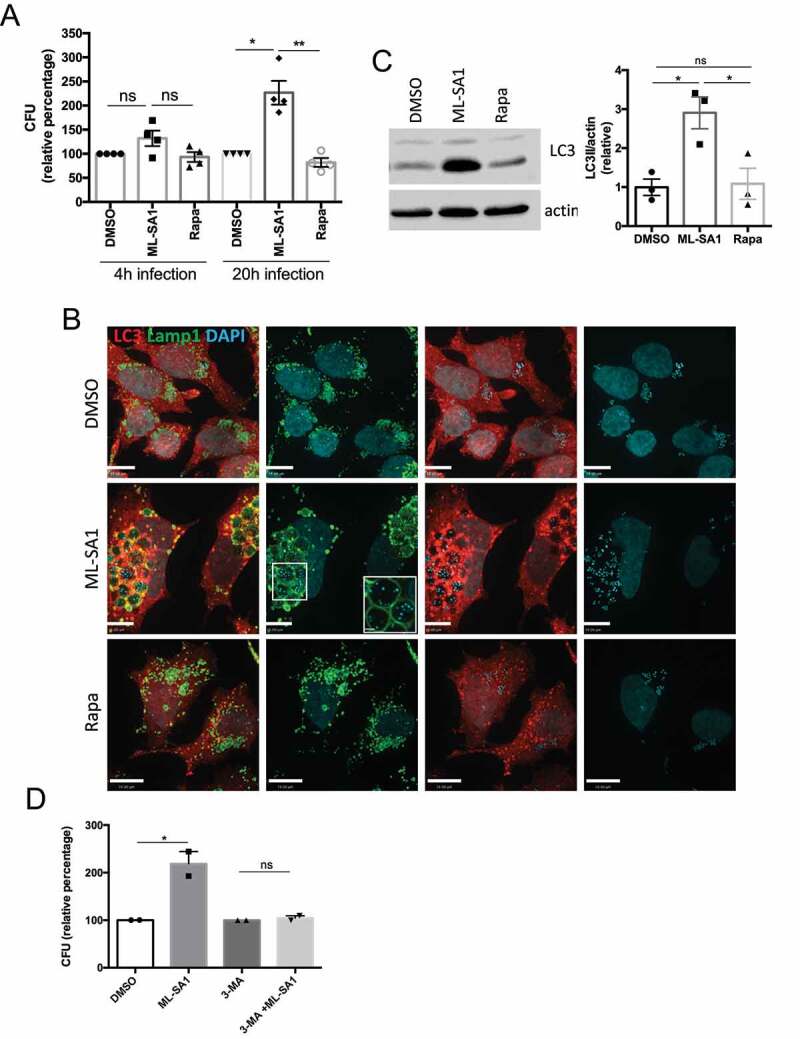
Effect of MTOR and TRPML1 pathways on *Sa* intracellular survival. (**A**) HeLa cells infected with *Sa* were incubated with gentamycin and treated for 4h or 20h with ML-SA1 (20 μM), rapamycin (Rapa, 100 nM) or vehicle control (DMSO). Intracellular bacteria were retrieved and CFU quantified. Graph shows relative percentage of CFU (mean +/- SEM of 4 independent experiments) considering 100 the bacteria retrieved from DMSO-treated cells. (**B**) LC3 and Lamp1 staining of cells infected and treated for 20h with ML-SA1 or rapamycin. Images in the separate channels are included at the right. (**C**) LC3 western blotting of *Sa*-infected cells treated as in (B) using actin as loading control. Graph shows quantification of LC3II normalized to actin (mean +/- SEM of 3 different experiments). (**D**) HeLa cells infected with *Lm* were treated with DMSO, ML-SA1 (20 μM), 3-methyladenine (3-MA) or 3-MA containing 20 μM ML-SA1 (3-MA + ML-SA1). Intracellular bacteria were retrieved and CFU quantified as in (**A**)

Triggering xenophagy in *Sa*-infected HeLa cells by TRPML1 activation or mTORC1 inhibition did not affect *Sa* intracellular survival at short times (4 hpi) (Figure 6A). However, ML-SA1 administration caused a large increase in CFU counts following long term infection (20 hpi), whereas rapamycin (100 nM) did not have an effect (Figure 6A). Although ML-SA1 produced a marked increase in the number of LC3 positive autophagosomes (Figure 6B) and increased LC3II levels by Western Blot (Figure 6C), most intracellular *Sa* were located in enlarged Lamp1-positive compartments at 20 hpi (Figure 6B and S8A,B). Indeed, ML-SA1 treatment increased the size and number of these compartments, which contained numerous bacteria (Figure S8B,C). Thus, the effect of TRPML1 on lysosomal biogenesis in the presence of Hla-expressing *Sa* promotes the formation of a Lamp1-positive intracellular reservoir that promotes *Sa* growth. Nevertheless, ML-SA1 effect depended on autophagy, as its administration was unable to increase *Sa* counts in the presence of 3-MA (Figure 6D).

### Effect of MTOR or TRPML1 pathways on bacteria that evade xenophagy recognition

*Shigella flexneri* (*Sf*) invades host cells and rapidly ruptures the endocytic vacuole to access the host cytosol, where the bacteria evade xenophagy and undergo replication. *Sf* secretes IcsA/VirG to promote intracellular actin-based motility. However, IcsA is recognized by Atg5, which promotes xenophagy. To counteract this, *Sf* secretes IcsB which binds to IcsA/VirG thereby preventing recognition by Atg5 ^9,58^. Furthermore, IscB also prevents the formation of septin cages, necessary for the recruitment of ubiquitin and autophagy receptors p62 and NDP52 ^58^. Thus, intracellular growth of a mutant *Sf* lacking IscB is restricted by xenophagy ^58^.

**Figure 7.**
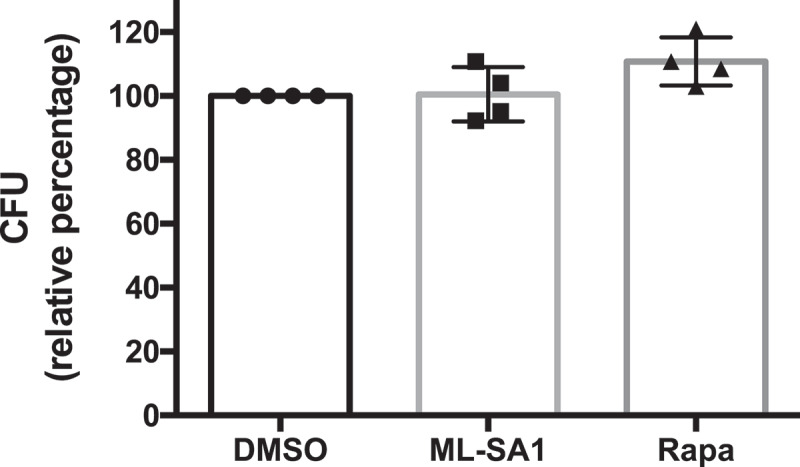
Effect of MTOR and TRPML1 pathways on *Sf* intracellular survival. (**A**) HeLa cells infected with *Sf* were incubated with gentamycin and treated for 4h with ML-SA1 (20 μM), rapamycin (Rapa, 100 nM) or vehicle control (DMSO). Intracellular bacteria were retrieved and CFU quantified. Graph shows relative percentage of CFU (mean +/- SEM of 4 independent experiments) considering 100 the bacteria retrieved from DMSO-treated cells.

Given the ability of the bacterium to hide from the xenophagy machinery, we suspected that neither inhibiting mTORC1 with rapamycin (nM), nor activating TRPML1 by ML-SA1 would enhance *Sf* killing through xenophagy. Indeed, there was no difference in *Sf* viability in cells treated with ML-SA1 or rapamycin (100 nM) in comparison with control cells (Figure 7).

## Discussion

Autophagy is a key player in innate immunity. In fact, in vivo models of infection highlight the role of xenophagy in intracellular pathogen clearance, as knocking down essential autophagy genes, like Atg5 or Atg16L1, increases bacterial load in mice infected with *Salmonella* ^59,60^. The importance of both MTOR and TRPML1-TFEB pathways in clearing intracellular bacteria is underscored by the numerous ways in which pathogens manipulate these pathways to establish a successful infection. For example, *Hp* toxin VacA inhibits TRPML1^41,43^, *St* maintains TFEB in an inactive state in the cytoplasm^54^ and mTORC1 active on the SCV^36^, and *Sf* effector OspB activates mTORC1 to inhibit autophagy and induce cell proliferation providing more cells for the bacteria to infect ^61^.

Targeting the xenophagy pathway as a therapeutic strategy during infection with intracellular pathogens is an attractive concept ^10,11^. However, due to the myriad ways that intracellular pathogens manipulate this pathway, a precision approach is likely required. Therefore, in this study we investigated the effect of xenophagy induction by mTORC1 or TRPML1-TFEB on bacterial survival during infection with prototypic intracellular pathogens that are either susceptible to, evade or depend on autophagy for intracellular survival.

Rapamycin is commonly employed to induce autophagy experimentally, an effect that is mechanistically attributed to its inhibitory effect on mTORC1 ^32,33^. However, as rapamycin also activates TRPML1 ^22^, studies performed with micromolar doses of rapamycin may have led to an overestimation of the beneficial effects of mTORC1 inhibition on autophagy without considering the contribution of the TRPML1-TFEB axis. mTORC1 directly regulates the initiation of autophagy ^16–18^, whereas TRPML1-TFEB axis jointly triggers the biogenesis of both autophagosomes and lysosomes ^26^. The concomitant increase in lysosomal genes achieved by TRPML1 activation is especially relevant in pathological conditions with compromised lysosomal function, like LSD, neurodegenerative diseases, aging and intracellular pathogen infections ^6,7,21,25^. Thus, the effect of higher dose of rapamycin on TRPML1 activation may explain, in part, why micromolar doses of rapamycin are required for antiaging and neuroprotective effects ^62^. This may also explain why genetic ablation of MTOR does not phenocopy rapamycin, and why Torin, a much stronger mTORC1 inhibitor that does not activate TRPML1 ^22^, lacks protective effects on neuron death in in vivo models of Parkinson disease ^63^. Indeed, the activation of TRPML1 by higher dose rapamycin may provide an explanation for the studies by Zullo et at. (2014), who reported that μM concentrations of rapamycin were required to mediate *Mycobacterium smegmatis* killing, effect that still occurred in LC3B- and Atg5-deficient bone marrow derived macrophages ^64^.

**Figure 8.**
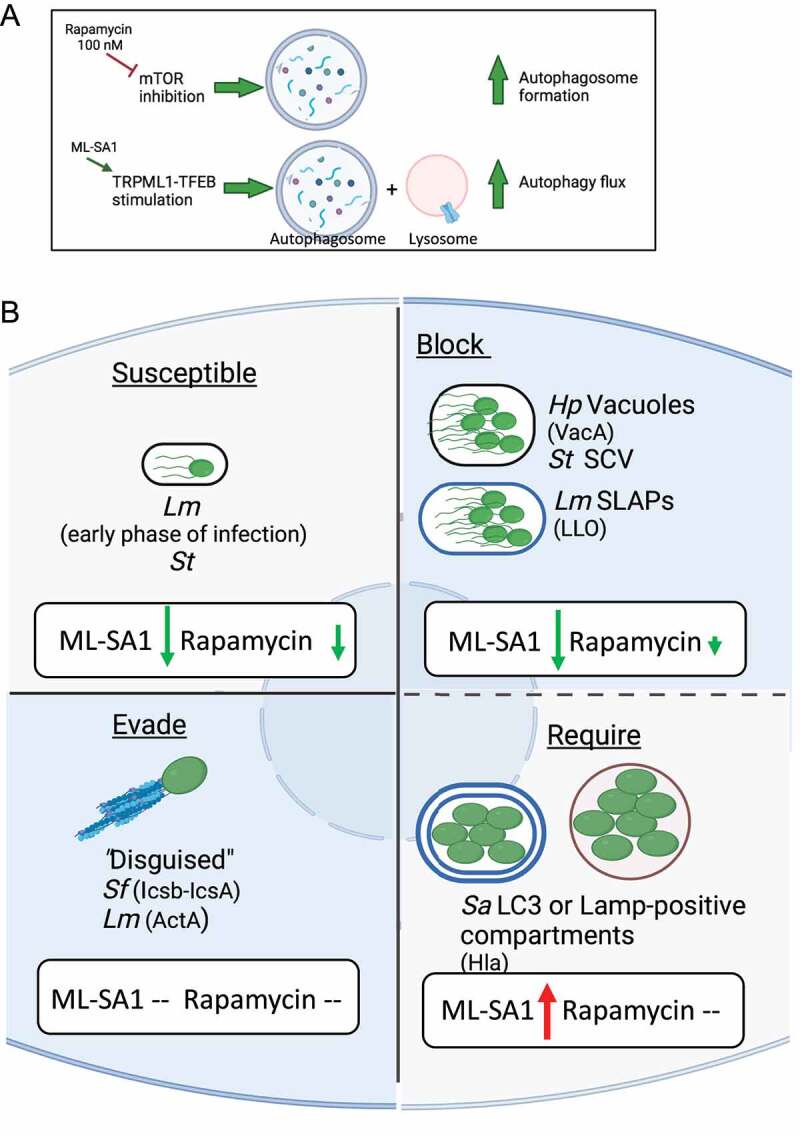
(**A**) Model for the differential effect of 100 nM rapamycin and ML-SA1 on autophagy pathway. (**B**) Working model illustrating the effect of inducing autophagy by TRPML1 activation (ML-SA1) or mTORC1 inhibition (Rapamycin) on bacterial growth for pathogens that are susceptible to, block, evade, or require autophagy for intracellular survival. Green arrow indicates a reduction, and red arrow an increase in intracellular bacterial growth; size of the arrow is proportional to the magnitude of the effect. –: no effect.

In this study, we first confirmed that micromolar doses of rapamycin indeed activate the TRPML1-TFEB axis. We next differentiated the specific effect of MTOR and TRPML1 pathways on autophagy by using rapamycin concentrations that inhibit mTORC1 but are unable to activate TRPML1; and the TRPML1 agonist ML-SA1 that does not affect mTORC1 activity. We showed that unlike the MTOR pathway, the TRPML1-TFEB axis strongly increased autophagic flux (Figure 8A).

During infection, the context in which the pathogen modulates xenophagy impacted the effects of activation of xenophagy pathway on intracellular survival. Not surprisingly, when the effect of rapamycin was limited to mTORC1 inhibition, the impact on bacterial clearance was limited as well. For pathogens susceptible to xenophagy, TRPML1 activation by ML-SA1 exhibited a stronger antibacterial effect than mTORC1 inhibition by rapamycin at nM doses (Figure 8B). We found that TRPML1 activation enhanced the capture of pathogens by xenophagy and restored endolysosmal vesicular trafficking, thereby increasing the lysosome population. Thus, unlike mTORC1 inhibition, TRPML1 activation increased bacterial killing of *Hp* in vacuoles, *Lm* in SLAPs and *St* in SCV/Sifs. However, in the case of *Sa*, TRPML1 activation increased the formation of intracellular bacterial reservoir, potentiating bacterial growth (Figure 8B).

Notably, for some pathogens a significant effect of TRPML1-TFEB axis on bacterial growth was identified at longer time points than the commonly employed time frames used in vitro studies of infection. For example, after prolonged treatment, ML-SA1 decreased *St* CFU and enhanced *Sa* numbers. Therefore, the timing of infection is another variable to be evaluated when studying the response of intracellular infection to drugs. In these studies, we employed a reductionist approach using in vitro model systems to delineate the impact of TRPML1-TFEB versus mTORC1-mediated xenophagy on bacterial survival. However, future studies will be required to determine the impact of these pathways during infection in vivo.

In summary, using prototypical pathogens that are susceptible to, evade or require autophagy for intracellular survival, we showed that the outcome of inducing xenophagy on pathogen growth is highly variable; and is strongly influenced by the host cell pathways used to activate xenophagy. While autophagy is an attractive host-directed therapeutic to target intracellular pathogens without increasing antibacterial resistance, a deep understanding of the host-pathogen interaction and the autophagy modulator used is required to properly curb infection. We suggest that a precision approach to modulation of xenophagy for therapeutic purposes is warranted.

## Materials and methods

### Cell lines and bacterial strains

HeLa cells and TRPML1 KO and wild-type MEFs were grown in Dulbecco’s Modified Eagle’s Medium (DMEM; Wisent Inc., 319-005-CL) supplemented with 10% fetal bovine serum (FBS, Wisent Inc., 080150), and human gastric epithelial AGS cells (ATCC, CRL-1739) were cultured in Ham’s F-12 (Wisent Inc., 318-021-CL) containing 10% FBS. All cell lines were maintained in 5% CO_2_ at 37**°**C. *H. pylori* strain 60190 (VacA [*s1i1m1*], CagA) and the isogenic VacA- mutant were provided by R. Peek Jr (Vanderbilt University School of Medicine, Nashville); *S*. Typhimurium, *L. monocytogenes* and *S. flexneri* were a kind gift from D. Philpott (University of Toronto, Toronto); and *S. aureus* was obtained from M. Terebiznik (University of Toronto). *St* and *Sa* were grown from single colony in LB broth (Fisher Scientific BP1426-500), *Lm* in brain heart infusion broth medium (BHI; BBL BD, 211059) supplemented with chloramphenicol (Bioshop, CLR201) and *Sf* in tryptic soy broth (TSB; BD, Df0373-17-3) containing spectinomycin (Sigma Aldrich, S0692). *Hp* was grown on Columbia Blood agar plates (Oxoid) for 2-3 days and then transferred to Brucella broth (Fluka, B3051) supplemented with 10% FBS for 16-24h. *Hp* cultures were maintained at 37°C under microaerophilic conditions (5% O_2_, 10% CO_2_, 85% N_2_). To prepare VacA+ or VacA- concentrated conditioned culture media (CCMS), broth cultures were grown to optical density (OD) of 1.0 at 600 nm, and the supernatants concentrated 10 times using a 30 kDa-cut off Amicon Ultra centrifugal filters (Ultracel, Millipore, UFC903024).

### Cell treatments

Cells plated on glass coverslips for immunofluorescence studies, or on 6-well plates for Western Blotting, were subjected to starvation (briefly washed twice in PBS and cultured in Earle’s balanced salt solution [Gibco, 24010-043]), or treated with ML-SA1 (Sigma Aldrich, SML0627; 20 μM), Rapamycin (Stemcell Technologies, 73362; 100 nM or 20 μM), Torin (EMD Millipore Corp., 475991; 250 nM or 1 μM), Bafilomycin A1 (Cayman Chemical company, 11038; 100 nM) or same volume of DMSO (Sigma Aldrich, D4540) as vehicle control. Alternatively, ML-SA1 was prepared in cell culture medium containing 5 mM 3-methyladenine (3-MA, Sigma Aldrich, M9281). When indicated, VacA CCMS (1X final concentration) was added, bacterial infections were performed or eGFPmRFP-LC3 construct transfected into HeLa cells using Lipofectamine 2000 prior to the treatment administrations.

### Cell Infections

For *Hp* infections, overnight bacterial cultures were pelleted and *Hp* resuspended to an OD of 1 in cell culture media (OD 600 nm of 1= 2x10^8^ bacteria/ml). Bacteria were added to AGS cells at a multiplicity of infection (moi) of 50 for 4h, unattached bacteria were removed by phosphate buffered saline (PBS, Wisent Inc. 311-010-CL) washes, and cell culture medium supplemented with 100 μg/ml gentamycin (Wisent Inc., 450-135-XL) was added for 1h. Monolayers were washed PBS, and gentamycin reduced to 10 μg/ml in the presence of the indicated treatments for the remaining infection time. Intracellular bacteria were retrieved by lysing the infected cells in 0.1% saponin (Sigma Aldrich, S4521) in cell culture medium during 12 min; serial dilutions of *Hp* suspension were prepared in Brucella broth and drop-plated (50 μl drops) on Columbia Blood agar plates for CFU determinations.

For the other pathogens, overnight cultures were grown from single colonies and then sub-cultured (1%-2% inoculum) for an additional ~3h until reaching OD of 0.6-0.8. Bacteria were added to HeLa cells at a moi of 10 for *Sf*, 20 for *St*, and 50 for *Lm* and *Sa* for CFU determination; and moi of 100 for *St* and 50 for *Lm* for immunolabeling at 4hpi; and the plates spun down at 500g for 5min. Infections were performed for 30 min at 37**°**C, unattached bacteria removed by washing with PBS and medium containing high dose of gentamycin (50 μg/ml for *St* and 100 μg/ml for *Sa, Lm* and *Sf*) was added for 40 min. After 4 washes with PBS, medium containing low dose of gentamycin (5 μg/ml for *St* and 10 μg/ml for *Sa, Lm* and *Sf*) in the presence of the indicated treatment was added for additional 4h or ~22h. Intracellular bacteria were retrieved by lysing the infected cells in 0.1% Triton X100 (Sigma Aldrich, X-100) in PBS; serial dilutions were prepared in PBS and drop-plated (10 μl drops) on the corresponding agar plates for CFU determinations.

### Western blotting

Cell were lysed in RIPA buffer, samples run on SDS-PAGE gels and transferred to nitrocellulose membranes (BioRad, 1620115). Membranes were blocked in 5% skimmed milkor 5% BSA (for phospho-p70S6K and phospho-mTOR antibodies) dissolved in Tris buffer saline containing 0.1% Tween 20 (BioShop, TWN510.100) and primary antibodies were incubated over night at 4°C in blocking solution. HRP-conjugated secondary antibodies were then added for 1-2h at room temperature, signals were obtained using chemiluminescence substrate (Santa Cruz, SC-2048) and densitometry analyses were performed using Li-Cor Odyssey Fc imaging system. Primary antibodies utilized in this study include LC3 (Novus Biologicals, NB600-1384; 1:1000), TFEB (Cell Signaling Technologies, 4240; 1:1000), actin (Sigma Aldrich clone AC-15, A5441; 1:5000), P-p70S6K (Cell Signaling, 9205; 1:1000), p70S6K (Cell Signaling, 9202; 1:1000), P-mTOR (Cell Signaling, 2971; 1:1000) and mTOR (Cell Signaling, 2972; 1:1000). Secondary antibodies used were HRP-conjugated goat anti-rabbit (Cedarlane, 111-035-144; 1:5000) and HRP-conjugated goat anti-mouse (Cedarlane,111-035-003; 1:5000).

### Immunofluorescence microscopy

Cells were fixed in 4% paraformaldehyde (Electron Microscopy Sciences, 15710) for 20 min, permeabilized with ice-cold methanol for 15 min (LC3, TFEB and Lamp1 staining) or 0.25% Triton X100 in PBS (p62, P-p62 and P-TBK1 staining) and blocked with 5% BSA (Sigma Aldrich, A7906) prepared in PBS for 1h. Incubations with primary antibodies resuspended in blocking buffer were then performed overnight at 4°C, secondary antibodies were added for 1h at room temperature, and nuclei/bacteria visualized by DAPI (4’,6’-diamidino-2-phenylindole, 1μg/ml Thermo Fisher Scientific, D1306) staining. Coverslips were mounted using Dako Fluorescence Mounting Medium (Agilent Technologies, S3023). Primary antibodies used include LC3 (1:200), TFEB (1:500), Lamp1 (Developmental Studies Hybridoma bank, H4A3; 1:75), p62 (abcam, ab564116; 1:50), Phospho-p62(Ser403) (MBL, D343-3; 1:100) and Phospho-TBK1(Ser172) (Cell Signaling Technologies, 5483; 1:100). Alexa-Fluor-568-conjugated goat-anti-rabbit and Alexa-Fluor-488-conjugated goat-anti-mouse or goat anti-rat (Thermo Fisher Scientific A-11036 and A-11029 respectively; both 1:1000) were used as secondary antibodies. All images were acquired using a Quorum spinning-disc confocal microscopy, controlled by Volocity acquisition software (Perkin Elmer). For quantification purposes, images were captured in randomly selected fields by focusing on DAPI signal, or randomly selected transfected cells.

### Statistical analysis

The sample size (n) of each experiment is included in the corresponding figure legend. GraphPad Prism software was used for all statistical analysis. All histograms present the mean +/- standard error of the mean (represented as error bars) and include the scatter plot data. A one-way analysis of variance with multiple comparisons (Tukey correction) was used for comparisons among groups. Statistical significance was set at *P*<0.05.

## Supplementary Material

Supplemental Material

## References

[1] Bloom DE, Cadarette D. Infectious disease threats in the twenty-first century: Strengthening the global response. *Front Immunol*. 2019;10 (MAR):1–12. doi:10.3389/fimmu.2019.00549 PMC6447676 30984169

[2] Murray CJ, Ikuta KS, Sharara F, et al. Global burden of bacterial antimicrobial resistance in 2019: a systematic analysis. *Lancet*. 2022;399(10325):629–26. doi:10.1016/S0140-6736(21)027240)027240 35065702 PMC8841637

[3] Kaufmann SHE, Dorhoi A, Hotchkiss RS, Bartenschlager R. Host-directed therapies for bacterial and viral infections. *Nat Rev Drug Discov*. 2018;17(1):35–56. doi:10.1038/nrd.2017.162 28935918 PMC7097079

[4] Bergman P, Raqib R, Rekha RS, Agerberth B, Gudmundsson GH. Host Directed Therapy Against Infection by Boosting Innate Immunity. *Front Immunol*. 2020;11(June). doi:10.3389/fimmu.2020.01209 PMC7304486 32595649

[5] Kumar Y, Valdivia RH. Leading a Sheltered Life: Intracellular Pathogens and Maintenance of Vacuolar Compartments. *Cell Host Microbe*. 2009;5(6):593–601. doi:10.1016/j.chom.2009.05.014 19527886 PMC2716004

[6] Gruenberg J, Van Der Goot FG. Mechanisms of pathogen entry through the endosomal compartments. *Nat Rev Mol Cell Biol*. 2006;7(7):495–504. doi:10.1038/nrm1959 16773132

[7] Sachdeva K, Sundaramurthy V. The Interplay of Host Lysosomes and Intracellular Pathogens. *Front Cell Infect Microbiol*. 2020;10 (November):1–13. doi:10.3389/fcimb.2020.595502 PMC7714789 33330138

[8] Levine B, Kroemer G. Autophagy in the Pathogenesis of Disease. *Cell*. 2008;132(1):27–42. doi:10.1016/j.cell.2007.12.018 18191218 PMC2696814

[9] Mao K, Klionsky DJ. Xenophagy: A battlefield between host and microbe, and a possible avenue for cancer treatment. *Autophagy*. 2017;13(2):223–224. doi:10.1080/15548627.2016.1267075 28026986 PMC5324837

[10] Hu W, Chan H, Lu L, et al. Autophagy in intracellular bacterial infection. *Semin Cell Dev Biol*. 2020;101(June2019): 41–50. doi:10.1016/j.semcdb.2019.07.014 31408699

[11] Riebisch AK, Mühlen S, Beer YY, Schmitz I. Autophagy—a story of bacteria interfering with the host cell degradation machinery. *Pathogens*. 2021;10(2):1–24. doi:10.3390/pathogens10020110 PMC7911818 33499114

[12] Huang J, Brumell JH. Bacteria–autophagy interplay: a battle for survival. *Nat Rev Microbiol*. 2014;12(2):101–114. doi:10.1038/nrmicro3160 24384599 PMC7097477

[13] Cemma M, Brumell JHH. Interactions of Pathogenic Bacteria with Autophagy Systems. *Curr Biol*. 2012;22(13):R540–R545. doi:10.1016/j.cub.2012.06.001 22790007

[14] Reggio A, Buonomo V, Grumati P. Eating the unknown: Xenophagy and ER-phagy are cytoprotective defenses against pathogens. *Exp Cell Res*. 2020;396(1):112276. doi:10.1016/j.yexcr.2020.112276 32918896 PMC7480532

[15] Mitchell G, Isberg RR. Innate Immunity to Intracellular Pathogens: Balancing Microbial Elimination and Inflammation. *Cell Host Microbe*. 2017;22(2):166–175. doi:10.1016/j.chom.2017.07.005 28799902 PMC5562164

[16] Kim J, Kundu M, Viollet B, Guan KL. AMPK and mTOR regulate autophagy through direct phosphorylation of Ulk1. *Nat Cell Biol*. 2011;13(2):132–141. doi:10.1038/ncb2152 21258367 PMC3987946

[17] Nao Hosokawa †, Taichi Hara †, Takeshi Kaizuka CK, et al. Nutrient-dependent mTORC1 Association with the ULK1–Atg13–FIP200 Complex Required for Autophagy. *Mol Biol Cell*. 2009;20:1981–1991. doi:10.1091/mbc.E08 19211835 PMC2663915

[18] Liu GY, Sabatini DM. mTOR at the nexus of nutrition, growth, ageing and disease. *Nat Rev Mol Cell Biol*. 2020;21(4):183–203. doi:10.1038/s41580-019-0199-y 31937935 PMC7102936

[19] Kaur J, Debnath J. Autophagy at the crossroads of catabolism and anabolism. *Nat Rev Mol Cell Biol*. 2015;16(8):461–472. doi:10.1038/nrm4024 26177004

[20] Napolitano G, Ballabio A. TFEB at a glance. *J Cell Sci*. 2016;129(13):2475–2481. doi:10.1242/jcs.146365 27252382 PMC4958300

[21] Ballabio A. The awesome lysosome. *EMBO Mol Med*. 2016;8(2):73–76. doi:10.15252/emmm.201505966 26787653 PMC4734841

[22] Zhang X, Chen W, Gao Q, et al. Rapamycin directly activates lysosomal mucolipin TRP channels independent of mTOR. *PLoS Biol*. 2019;17(5):e3000252. doi:10.1371/journal.pbio.3000252 31112550 PMC6528971

[23] Medina DL, Fraldi A, Bouche V, et al. Transcriptional activation of lysosomal exocytosis promotes cellular clearance. *Dev Cell*. 2011;21(3):421–430. doi:10.1016/j.devcel.2011.07.016 21889421 PMC3173716

[24] Spampanato C, Feeney E, Li L, et al. Transcription factor EB (TFEB) is a new therapeutic target for Pompe disease. *EMBO Mol Med*. 2013;5(5):691–706. doi:10.1002/emmm.201202176 23606558 PMC3662313

[25] Settembre C, Fraldi A, Medina DL, Ballabio A. Signals from the lysosome: a control centre for cellular clearance and energy metabolism. *Nat Rev Mol Cell Biol*. 2013;14(5):283–296. doi:10.1038/nrm3565 23609508 PMC4387238

[26] Settembre C, Di Malta C, Polito VA, et al. TFEB Links Autophagy to Lysosomal Biogenesis. *Science (80-)*. 2011;332(6036):1429–1433. doi:10.1126/science.1204592.TFEB PMC3638014 21617040

[27] Marco Sardiello, Michela Palmieri, Alberto di Ronza, Diego Luis Medina, Marta Valenza, Vincenzo Alessandro Gennarino, Chiara Di Malta, Francesca Donaudy, Valerio Embrione, Roman S. Polishchuk. A Gene Network Regulating Lysosomal Biogenesis and Function. *Science*. 2009;235 (July):473–478. 10.1126/science.1174447 19556463

[28] Puertollano R, Ferguson SM, Brugarolas J, Ballabio A. The complex relationship between TFEB transcription factor phosphorylation and subcellular localization. *EMBO J*. 2018;37(11):1–12. doi:10.15252/embj.201798804 29764979 PMC5983138

[29] Medina DL, Di Paola S, Peluso I, et al. Lysosomal calcium signalling regulates autophagy through calcineurin and TFEB. *Nat Cell Biol*. 2015;17(3):288–299. doi:10.1038/ncb3114 25720963 PMC4801004

[30] Venkatachalam K, Wong CO, Zhu MX. The role of TRPMLs in endolysosomal trafficking and function. *Cell Calcium*. 2015;58(1):48–56. doi:10.1016/j.ceca.2014.10.008 25465891 PMC4412768

[31] Cheng X, Shen D, Samie M, Xu H. Mucolipins: Intracellular TRPML1-3 channels. *FEBS Lett*. 2010;584(10):2013–2021. doi:10.1016/j.febslet.2009.12.056 20074572 PMC2866799

[32] Ruan B, Pong K, Jow F, et al. Binding of rapamycin analogs to calcium channels and FKBP52 contributes to their neuroprotective activities. *Proc Natl Acad Sci U S A*. 2008;105(1):33–38. doi:10.1073/pnas.0710424105 18162540 PMC2224212

[33] Li J, Kim SG, Blenis J. Rapamycin: One drug, many effects. *Cell Metab*. 2014;19(3):373–379. doi:10.1016/j.cmet.2014.01.001 24508508 PMC3972801

[34] Birmingham CL, Canadien V, Gouin E, et al. Listeria monocytogenes evades killing by autophagy during colonization of host cells. *Autophagy*. 2007;3(5):442–451. doi:10.4161/auto.4450 17568179

[35] Lapaquette P, Bringer MA, Darfeuille-Michaud A. Defects in autophagy favour adherent-invasive Escherichia coli persistence within macrophages leading to increased pro-inflammatory response. *Cell Microbiol*. 2012;14(6):791–807. doi:10.1111/j.1462-5822.2012.01768.x 22309232

[36] Tattoli I, Sorbara MT, Vuckovic D, et al. Amino acid starvation induced by invasive bacterial pathogens triggers an innate host defense program. *Cell Host Microbe*. 2012;11(6):563–575. doi:10.1016/j.chom.2012.04.012 22704617

[37] Gutierrez MG, Master SS, Singh SB, Taylor GA, Colombo MI, Deretic V. Autophagy is a defense mechanism inhibiting BCG and Mycobacterium tuberculosis survival in infected macrophages. *Cell*. 2004;119(6):753–766. doi:10.1016/j.cell.2004.11.038 15607973

[38] Shahnazari S, Namolovan A, Mogridge J, Kim PK, Brumell JH. Bacterial toxins can inhibit host cell autophagy through cAMP generation. *Autophagy*. 2011;7(9):957–965. doi:10.4161/auto.7.9.16435 21606683

[39] Liu Y, Yang F, Zou S, Qu L. Rapamycin: A bacteria-derived immunosuppressant that has anti-atherosclerotic effects and its clinical application. *Front Pharmacol*. 2019;9 (JAN):1–15. doi:10.3389/fphar.2018.01520 PMC6330346 30666207

[40] Chen C-C, Keller M, Hess M, et al. A small molecule restores function to TRPML1 mutant isoforms responsible for mucolipidosis type IV. *Nat Commun*. 2014;5(May):1–10. doi:10.1038/ncomms5681 25119295

[41] Capurro MI, Prashar A, Jones NL. MCOLN1/TRPML1 inhibition - a novel strategy used by Helicobacter pylori to escape autophagic killing and antibiotic eradication therapy in vivo. *Autophagy*. 2020;16(1):169–170. doi:10.1080/15548627.2019.1677322 31599196 PMC6984606

[42] Prashar A, Capurro MI, Jones NL. Under the Radar: Strategies Used by Helicobacter pylori to evade host response. *Annu. Rev. Physiol*. 2022.84:485–506.doi:10.1146/annurev-physiol-061121-035930. 34672717

[43] Capurro MI, Greenfield LK, Prashar A, et al. VacA generates a protective intracellular reservoir for Helicobacter pylori that is eliminated by activation of the lysosomal calcium channel TRPML1. *Nat Microbiol*. 2019;4(8):1411–1423. doi:10.1038/s41564-019-0441-6 31110360 PMC6938649

[44] Hay N, Sonenberg N. Upstream and downstream of mTOR. *Genes Dev*. 2004;18(16):1926–1945. doi:10.1101/gad.1212704 15314020

[45] Mauvezin C, Neufeld TP. Bafilomycin A1 disrupts autophagic flux by inhibiting both. *Autophagy*. 2015;11(8):1437–1438. 26156798 10.1080/15548627.2015.1066957 PMC4590655

[46] Kimura S, Noda T, Yoshimori T. Dissection of the autophagosome maturation process by a novel reporter protein, tandem fluorescent-tagged LC3. *Autophagy*. 2007;3(5):452–460. doi:10.4161/auto.4451 17534139

[47] Li N, Tang B, Zhu ED, et al. Increased miR-222 in H. pylori-associated gastric cancer correlated with tumor progression by promoting cancer cell proliferation and targeting RECK. *FEBS Lett*. 2012;586(6):722–728. doi:10.1016/j.febslet.2012.01.025 22321642

[48] Raju D, Hussey S, Ang M, et al. Vacuolating cytotoxin and variants in Atg16L1 that disrupt autophagy promote helicobacter pylori infection in humans. *Gastroenterology*. 2012;142(5):1160–1171. doi:10.1053/j.gastro.2012.01.043 22333951 PMC3336037

[49] Terebiznik MR, Raju D, Vázquez CL, et al. Effect of Helicobacter pylori’s vacuolating cytotoxin on the autophagy pathway in gastric epithelial cells. *Autophagy*. 2009;5(3):370–379. doi:10.4161/auto.5.3.7663 19164948

[50] Birmingham CL, Smith AC, Bakowski MA, Yoshimori T, Brumell JH. Autophagy controls Salmonella infection in response to damage to the Salmonella-containing vacuole. *J Biol Chem*. 2006;281(16):11374–11383. doi:10.1074/jbc.M509157200 16495224

[51] Ganesan R, Hos NJ, Gutierrez S, et al. Salmonella Typhimurium disrupts Sirt1/AMPK checkpoint control of mTOR to impair autophagy. *PLoS Pathog*. 2017;13(2):1–22. doi:10.1371/journal.ppat.1006227 PMC5325604 28192515

[52] McGourty K, Thurston TLM, Matthews SA, Pinaud L, Mota LJ, Holden DW. Salmonella inhibits retrograde trafficking of mannose-6-phosphate receptors and lysosome function. *Science (80-)*. 2012;338:963–967. 10.1126/science.1227037 PMC6485626 23162002

[53] Zheng YT, Shahnazari S, Brech A, Lamark T, Johansen T, Brumell JH. The Adaptor Protein p62/SQSTM1 Targets Invading Bacteria to the Autophagy Pathway. *J Immunol*. 2009;183(9):5909–5916. doi:10.4049/jimmunol.0900441 19812211

[54] Ammanathan V, Mishra P, Chavalmane AK, et al. Restriction of intracellular Salmonella replication by restoring TFEB-mediated xenophagy. *Autophagy*. 2020;16(9):1584–1597. doi:10.1080/15548627.2019.1689770 31744366 PMC8386624

[55] Py BF, Lipinski MM, Yuan J. Autophagy limits Listeria monocytogenes intracellular growth in the early phase of primary infection. *Autophagy*. 2007;3(2):117–125. doi:10.4161/auto.3618 17204850

[56] Birmingham CL, Canadien V, Kaniuk NA, Steinberg BE, Higgins DE, Brumell JH. Listeriolysin O allows Listeria monocytogenes replication in macrophage vacuoles. *Nature*. 2008;451(7176):350–354. doi:10.1038/nature06479 18202661

[57] Mestre MB, Fader CM, Sola C, Colombo MI. α-hemolysin is required for the activation of the autophagic pathway in Staphylococcus aureus-infected cells. *Autophagy*. 2010;6(1):110–125. doi:10.4161/auto.6.1.10698 20110774

[58] Ogawa M, Yoshimori T, Suzuki T, Sagara H, Mizushima N, Saakawa C. Escape of intracellular shigella from autophagy. *Science (80-)*. 2005;307(5):727–731. doi:10.1093/bioinformatics/bti093 15576571

[59] Benjamin JL, Sumpter Jr. R, Levine B HL. Intestinal epithelial autophagy is essential for host defense Against Invasive Bacteria. *Cell Host Microbe*. 2013;13(6):723–734. doi:10.1016/j.chom.2013.05.004.Intestinal 23768496 PMC3755484

[60] Conway KL, Kuballa P, Song J-H, Patel KK, Castonero AB, Yilmaz OH, Jijon HB ZM. Atg16l1 i require for autophagy in intestinal epithelial cells and protection of mice from Salmonella infection. *Gastroenterology*. 2013;145(6):1–7. doi:10.1053/j.gastro.2013.08.035.Atg16l1 23973919 PMC3840157

[61] Lu R, Herrera BB, Eshleman HD, et al. Shigella Effector OspB Activates mTORC1 in a Manner That Depends on IQGAP1 and Promotes Cell Proliferation. *PLoS Pathog*. 2015;11(10):1–21. doi:10.1371/journal.ppat.1005200 PMC4608727 26473364

[62] Bové J, Martínez-Vicente M, Vila M. Fighting neurodegeneration with rapamycin: Mechanistic insights. *Nat Rev Neurosci*. 2011;12(8):437–452. doi:10.1038/nrn3068 21772323

[63] Malagelada C, Jin ZH, Jackson-Lewis V, Przedborski S, Greene LA. Rapamycin protects against neuron death in in vitro and in vivo models of Parkinson’s disease. *J Neurosci*. 2010;30(3):1166–1175. doi:10.1523/JNEUROSCI.3944-09.2010 20089925 PMC2880868

[64] Zullo AJ, Jurcic Smith KL, Lee S. Mammalian target of Rapamycin inhibition and mycobacterial survival are uncoupled in murine macrophages. *BMC Biochem*. 2014;15(1):1–10. doi:10.1186/1471-2091-15-4 PMC3937017 24528777

