# Supplementary material for: Survival of intracellular pathogens in response to mTORC1- or TRPML1-TFEB-induced xenophagy
Source: Autophagy Rep. 2023 Mar 19;2(1):2191918. doi: 10.1080/27694127.2023.2191918 (PMC12039413; doi:10.1080/27694127.2023.2191918)
Supplement: Supplemental Material [file KAUO_A_2191918_SM6084.zip › FigS1.pdf]

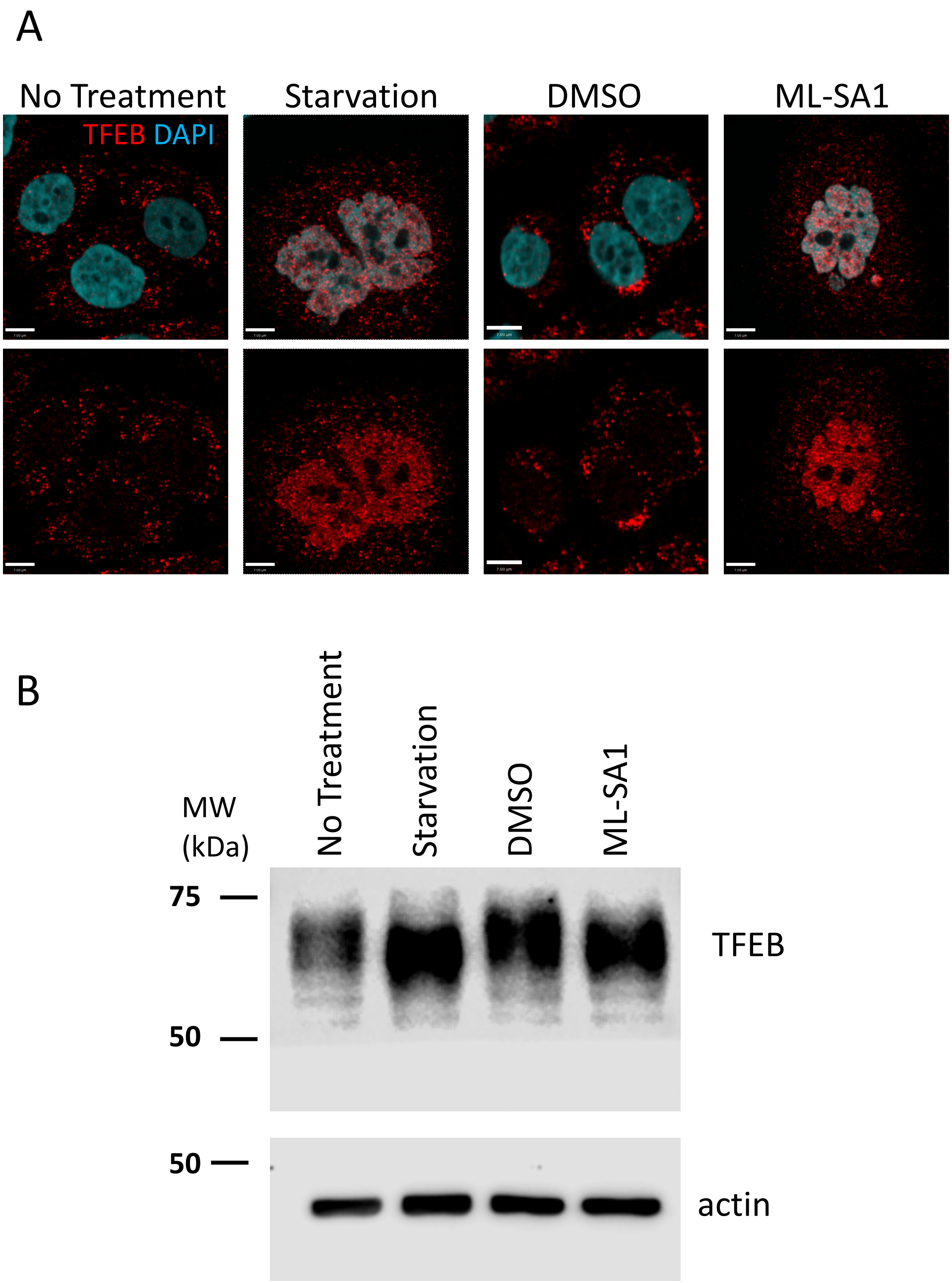

Supplementary Figure 1: (**A**) TFEB staining of AGS cells after 2 h starvation, treatment with ML-SA1 (20 µM) or vehicle control (DMSO). (**B**) TFEB western blotting of cell from (A) using actin as loading control. Molecular weight shift in TFEB band towards a lower molecular weight corresponds to dephosphorylated TFEB.
