# Supplementary material for: Survival of intracellular pathogens in response to mTORC1- or TRPML1-TFEB-induced xenophagy
Source: Autophagy Rep. 2023 Mar 19;2(1):2191918. doi: 10.1080/27694127.2023.2191918 (PMC12039413; doi:10.1080/27694127.2023.2191918)
Supplement: Supplemental Material [file KAUO_A_2191918_SM6084.zip › FigS2 revised.pdf]

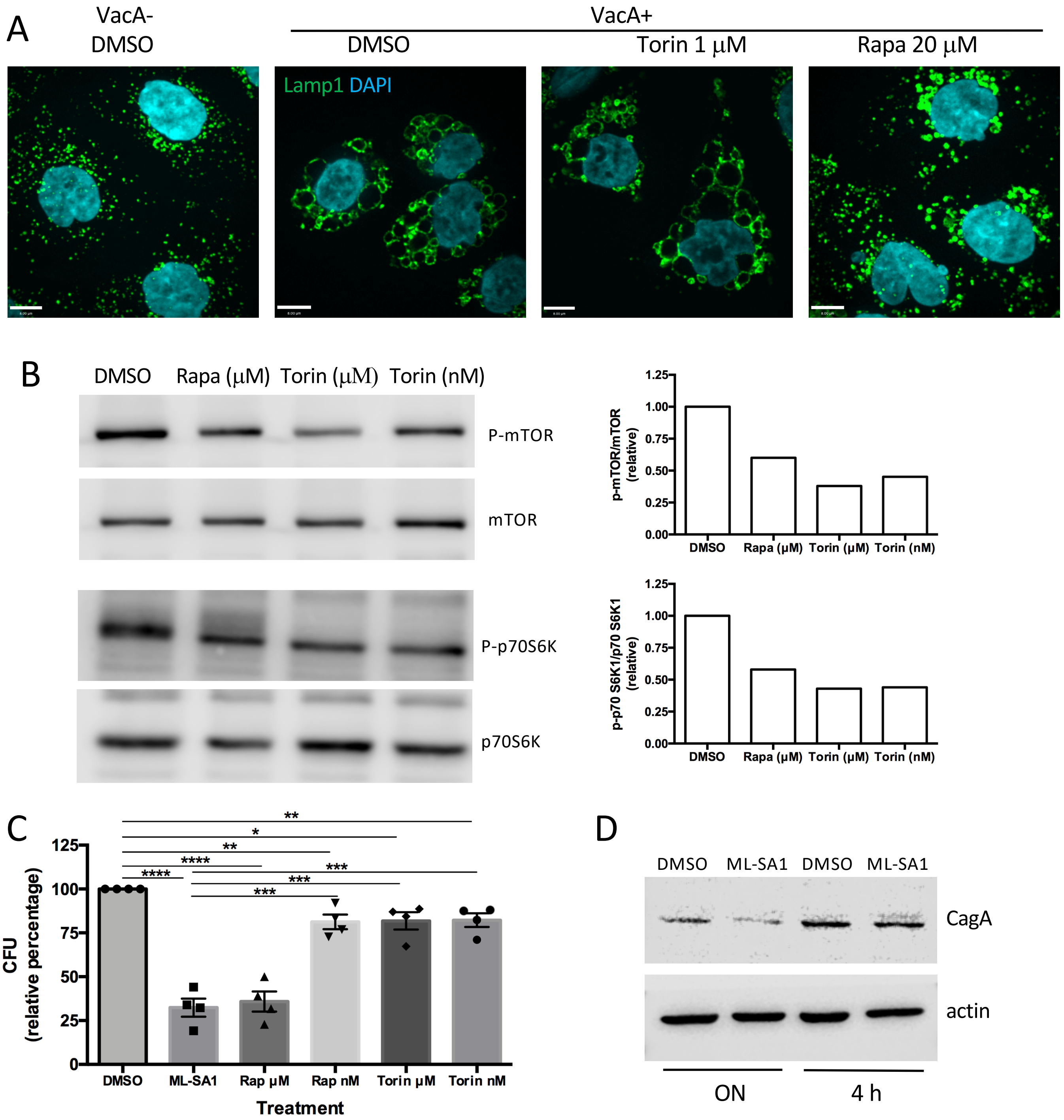

Supplementary Figure 2: (A) Lamp1 staining of AGS cells after 4h VacA- or VacA+ incubation followed by 3h of DMSO, Torin (1 µM) or rapamycin (Rapa, 20 µM) treatment. (**B**) Western blotting for mTOR and p70S6K phosphorylation after 3 h treatment with DMSO, Rapamycin (20 µM) or Torin (1 µM or 250 nM). *Right*: quantification of phospho-proteins normalized to total protein levels. (**C**) AGS cells infected with wild-type (VacA+) *Hp* were incubated with gentamycin to kill extracellular bacteria and treated with ML-SA1 (20 µM), rapamycin (20 µM or 100 nM), Torin (1 µM or 250 nM) or vehicle control (DMSO). Intracellular bacteria were retrieved and CFU quantified. Graph shows relative percentage of CFU (mean +/- SEM of 4 independent experiments) considering 100 the bacteria retrieved from DMSO-treated cells. (**D**) CagA western blotting of AGS cells infected with *Hp* and treated with DMSO or ML-SA1 (20 µM) overnight or during the last 4 h of infection. Actin was used as loading control.
