# Supplementary material for: Survival of intracellular pathogens in response to mTORC1- or TRPML1-TFEB-induced xenophagy
Source: Autophagy Rep. 2023 Mar 19;2(1):2191918. doi: 10.1080/27694127.2023.2191918 (PMC12039413; doi:10.1080/27694127.2023.2191918)
Supplement: Supplemental Material [file KAUO_A_2191918_SM6084.zip › FigS3.pdf]

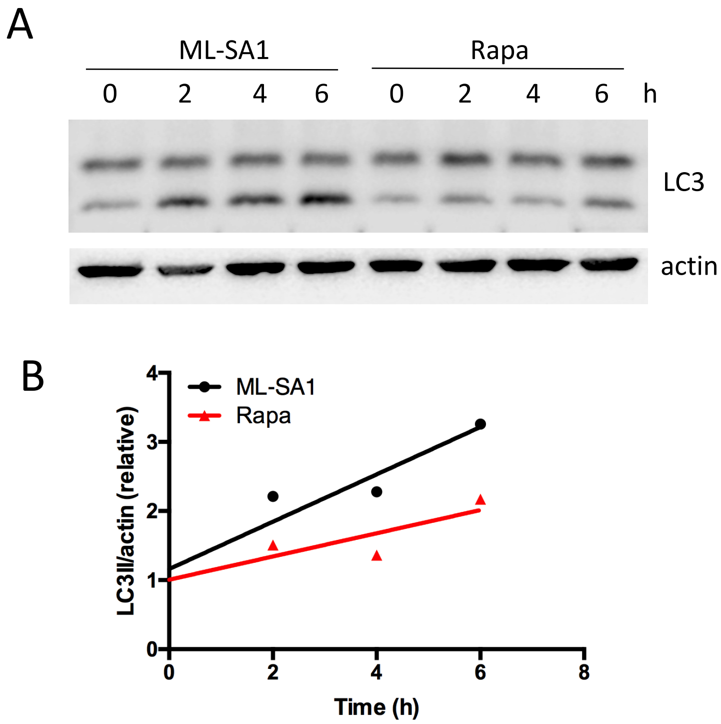

Supplementary Figure 3: (A) LC3 western blotting of HeLa cells treated with DMSO (0), ML-SA1 (20 µm) or rapamycin (Rapa, 100 nM) for the indicated time (in hours, h) using actin as loading control. (**B**) Graph shows the quantification of LC3II normalized to actin for each treatment, at each time. Lines (lineal regression) represent the time-dependent LC3II induction.
