# Supplementary material for: Survival of intracellular pathogens in response to mTORC1- or TRPML1-TFEB-induced xenophagy
Source: Autophagy Rep. 2023 Mar 19;2(1):2191918. doi: 10.1080/27694127.2023.2191918 (PMC12039413; doi:10.1080/27694127.2023.2191918)
Supplement: Supplemental Material [file KAUO_A_2191918_SM6084.zip › FigS4.pdf]

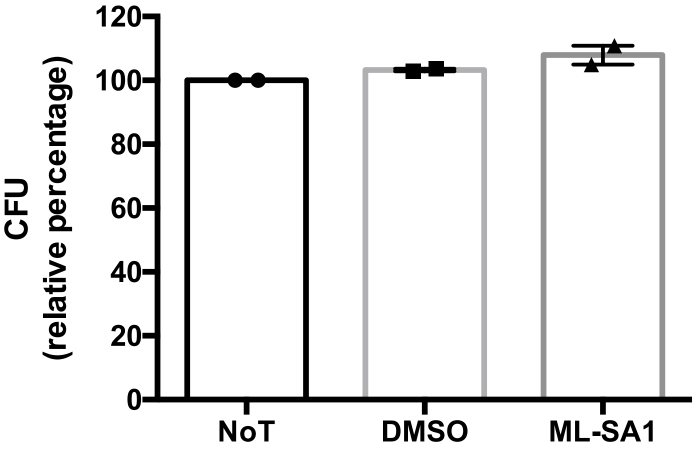

Supplementary Figure 4: Quantification of *Hp* growth after 4 h incubation with DMSO or ML-SA1 (20 μM). Graph shows the relative percentage of CFU compared with a control culture (NoT = no treatment) considered as 100%.
