# Supplementary material for: Survival of intracellular pathogens in response to mTORC1- or TRPML1-TFEB-induced xenophagy
Source: Autophagy Rep. 2023 Mar 19;2(1):2191918. doi: 10.1080/27694127.2023.2191918 (PMC12039413; doi:10.1080/27694127.2023.2191918)
Supplement: Supplemental Material [file KAUO_A_2191918_SM6084.zip › FigS5.pdf]

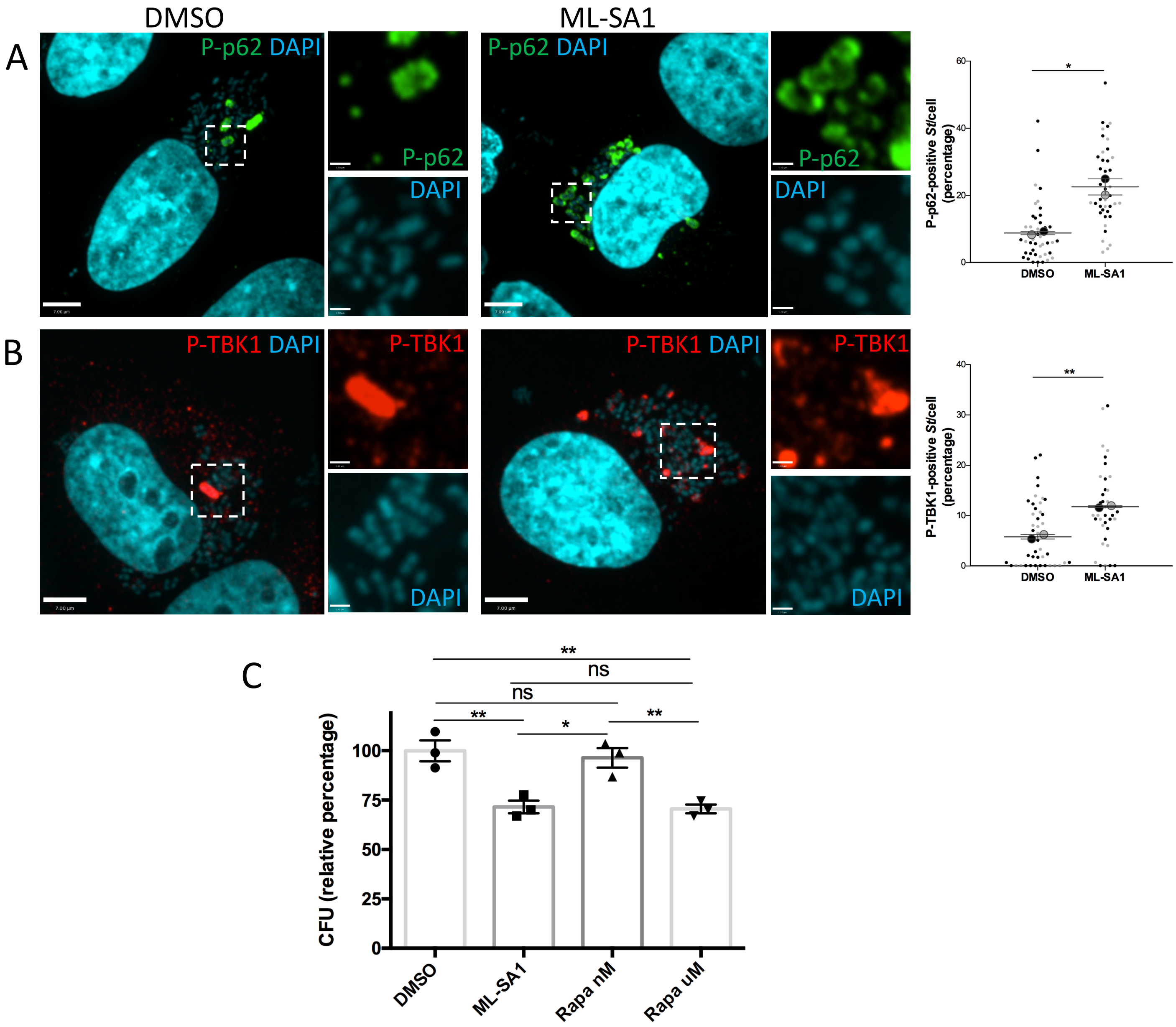

Supplementary Figure 5: (A) P-p62 staining of HeLa cells infected with *St* and treated with DMSO or ML-SA1 (20 µM) for 4h. DAPI staining used to visualize bacteria. Percentage of P-p62-positive bacteria on the right. Each color corresponds to 1 experiment, big symbols represent the mean and the small symbols the scatter dot plot/experiment. Lines show the mean +/- SEM. (**B**) P-TBK1 staining of HeLa cells infected with *St* and treated with DMSO or ML-SA1 (20 µM) for 4h. DAPI staining used to visualize bacteria. Percentage of P-TBK1-positive bacteria on the right plotted as in (**A**). (**C**) HeLa cells infected with *St* were incubated with gentamycin and treated for 23h with ML-SA1 (20µM), rapamycin 100 nM (Rapa nM), rapamycin 20 µM (Rapa uM) or vehicle control (DMSO). Intracellular bacteria were retrieved and CFU quantified. Graph shows relative percentage of CFU (mean +/- SEM of 3 independent experiments) considering 100% the bacteria retrieved from DMSO-treated cells.
