# Supplementary material for: Survival of intracellular pathogens in response to mTORC1- or TRPML1-TFEB-induced xenophagy
Source: Autophagy Rep. 2023 Mar 19;2(1):2191918. doi: 10.1080/27694127.2023.2191918 (PMC12039413; doi:10.1080/27694127.2023.2191918)
Supplement: Supplemental Material [file KAUO_A_2191918_SM6084.zip › FigS6.pdf]

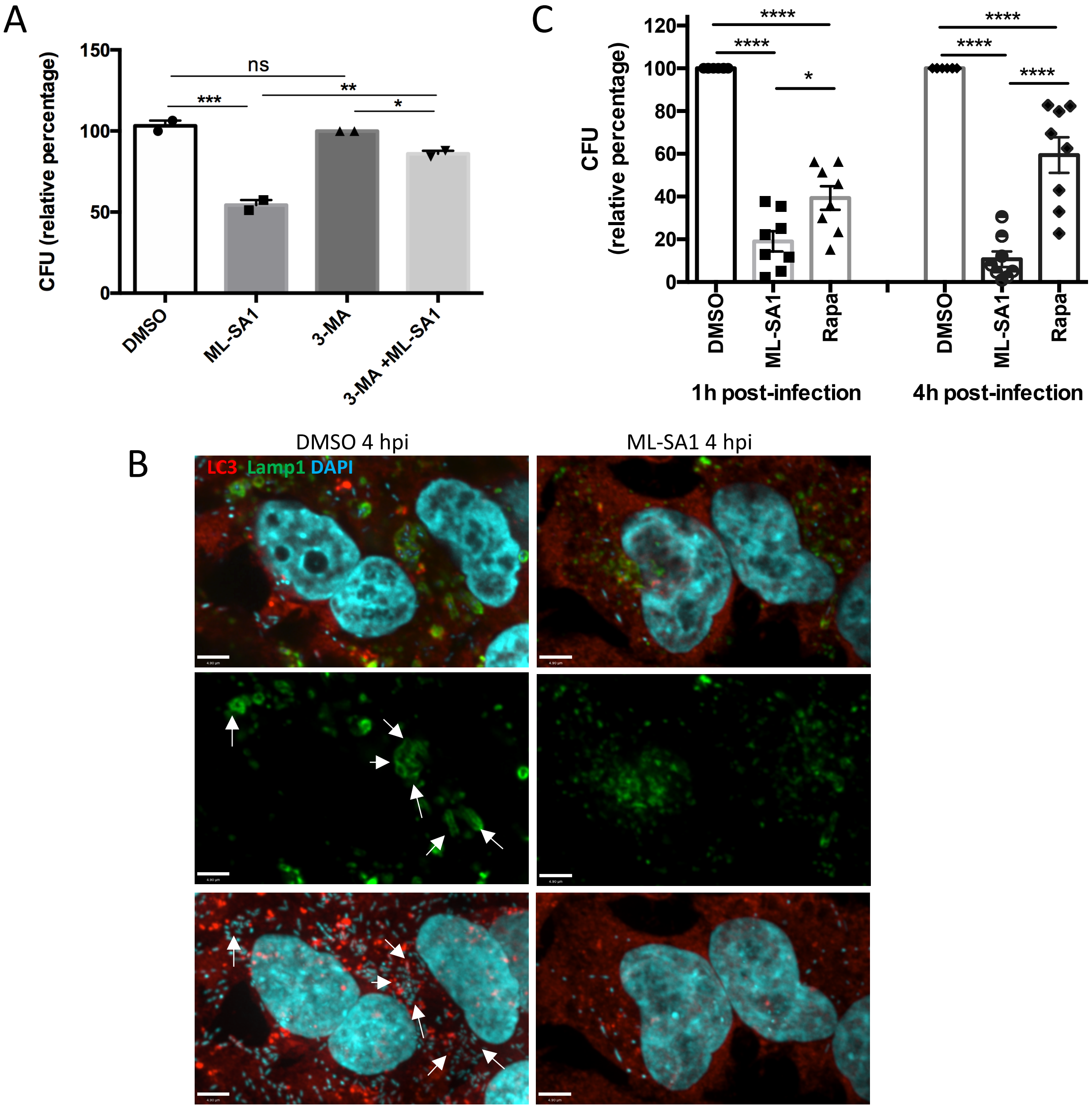

Supplementary Figure 6: **(A)** HeLa cells infected with *Lm* were incubated with gentamycin and treated with DMSO, ML-SA1 (20 µM), 3-methyladenine (3-MA, 5 mM) or 3-MA containing 20 µM ML-SA1 (3-MA + ML-SA1). Intracellular bacteria were retrieved and CFU quantified. Graph shows relative percentage of CFU considering 100 the bacteria retrieved from DMSO-treated cells. (**B**) LC3 and Lamp1 staining of cells infected with *Lm* and treated with DMSO or ML-SA1 (20 µM). Drugs were added 4 hpi and maintained for a total of 24 h infection. Arrows indicate SLAPs. (**C**) HeLa cells infected with *Lm* were incubated with gentamycin and treated with ML-SA1 (20 µM), rapamycin (Rapa, 100 nM) or vehicle control (DMSO). Treatments were added 1h or 4 h post-infection and maintained for a total of 24 h infection. Intracellular bacteria were retrieved and CFU quantified. Graph shows relative percentage of CFU (mean +/- SEM of 8 independent experiments), calculated as in (**A**).
