# Supplementary material for: Survival of intracellular pathogens in response to mTORC1- or TRPML1-TFEB-induced xenophagy
Source: Autophagy Rep. 2023 Mar 19;2(1):2191918. doi: 10.1080/27694127.2023.2191918 (PMC12039413; doi:10.1080/27694127.2023.2191918)
Supplement: Supplemental Material [file KAUO_A_2191918_SM6084.zip › FigS7.pdf]

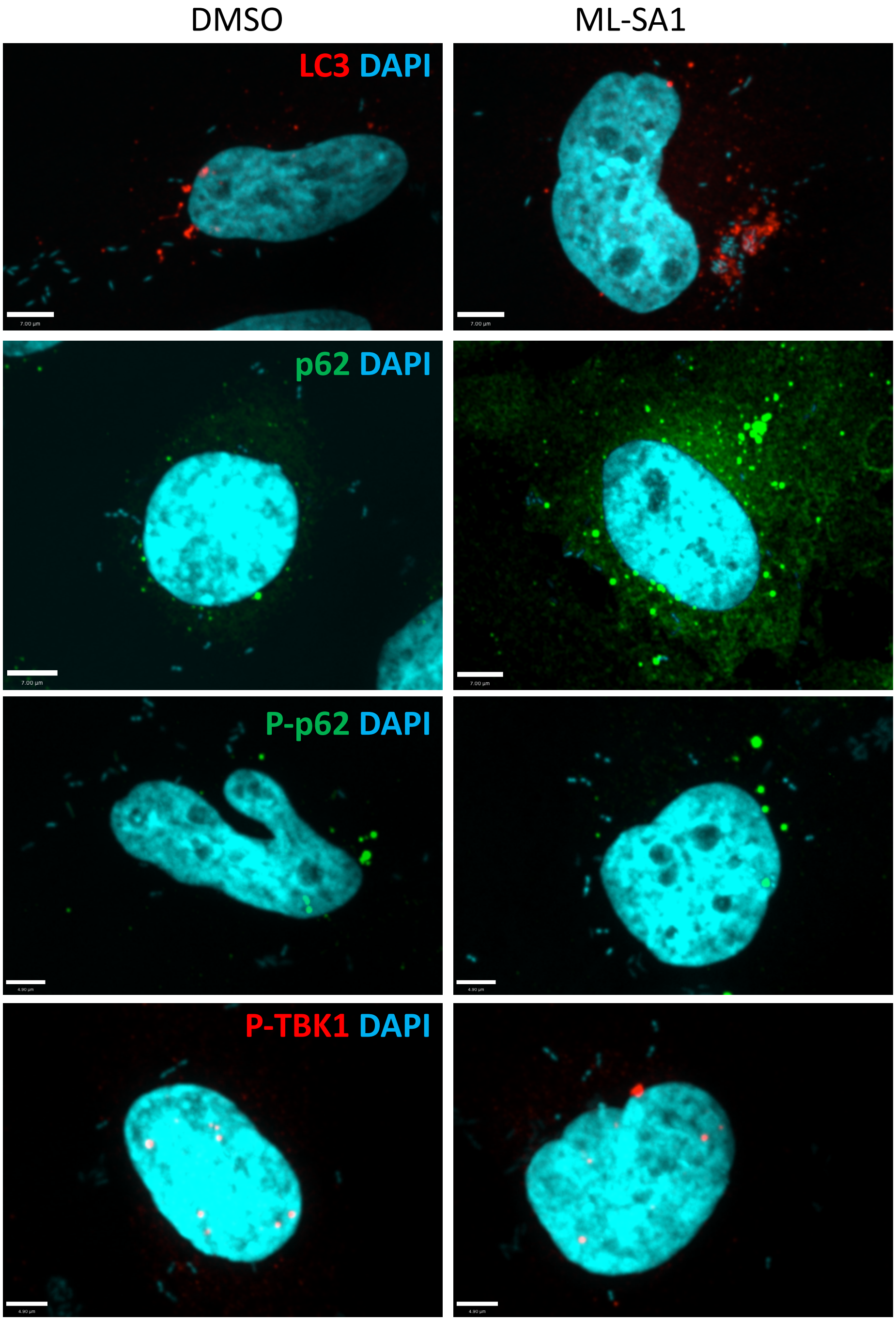

Supplementary Figure 7: LC3, p62, P-p62 and P-TBK1 staining of HeLa cells infected with *Lm* and treated with DMSO or ML-SA1 (20 μM) for 4h. DAPI staining used to visualize bacteria.
