# Supplementary material for: Survival of intracellular pathogens in response to mTORC1- or TRPML1-TFEB-induced xenophagy
Source: Autophagy Rep. 2023 Mar 19;2(1):2191918. doi: 10.1080/27694127.2023.2191918 (PMC12039413; doi:10.1080/27694127.2023.2191918)
Supplement: Supplemental Material [file KAUO_A_2191918_SM6084.zip › Revised FigS2.pdf]

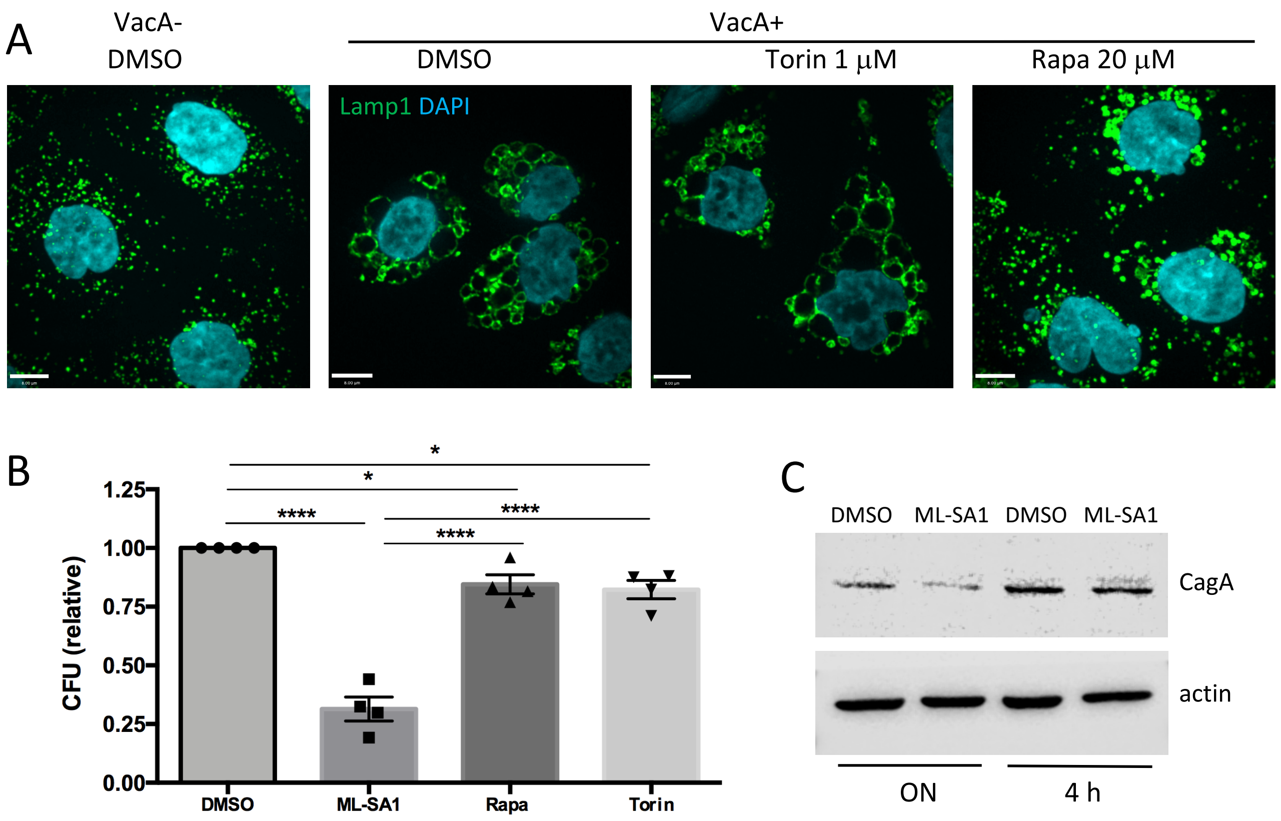

Supplementary Figure 2: (A) Lamp1 staining of AGS cells after 4h VacA- or VacA+ incubation followed by 3h of DMSO, Torin (1 μM) or rapamycin (Rapa, 20 μM) treatment. (**B**) AGS cells infected with wild-type (VacA+) *Hp* were incubated with gentamycin to kill extracellular bacteria and treated with ML-SA1 (20 μM), rapamycin (Rapa, 100 nM), Torin (1 μM) or vehicle control (DMSO). Intracellular bacteria were retrieved and CFU quantified. Graph shows relative CFU (mean +/- SEM of 4 independent experiments) considering 1 the bacteria retrieved from DMSO-treated cells. (**C**) CagA western blotting of AGS cells infected with *Hp* and treated with DMSO or ML-SA1 (20 μM) overnight or during the last 4 h of infection. Actin was used as loading control.
