# Supplementary material for: Survival of intracellular pathogens in response to mTORC1- or TRPML1-TFEB-induced xenophagy
Source: Autophagy Rep. 2023 Mar 19;2(1):2191918. doi: 10.1080/27694127.2023.2191918 (PMC12039413; doi:10.1080/27694127.2023.2191918)
Supplement: Supplemental Material [file KAUO_A_2191918_SM6084.zip › Supplementary Figures 1 to 6.pdf]

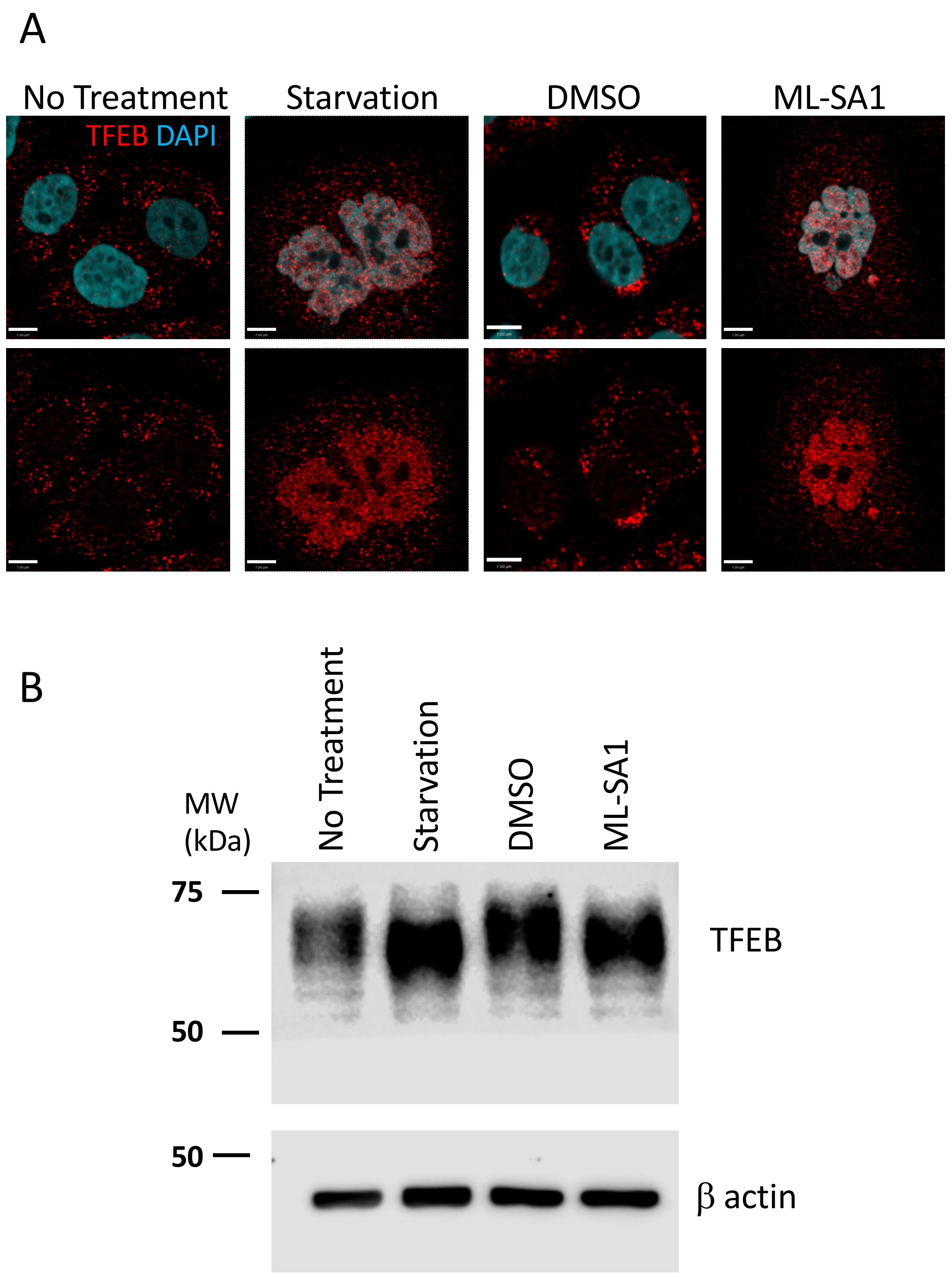

Supplementary Figure 1: (**A**) TFEB staining of AGS cells after 2 h starvation, treatment with ML-SA1 (20 mM) or vehicle control (DMSO). (**B**) TFEB western blotting of cell from (A) using b-actin as loading control. Molecular weight shift in TFEB band towards a lower molecular weight corresponds to dephosphorylated TFEB.

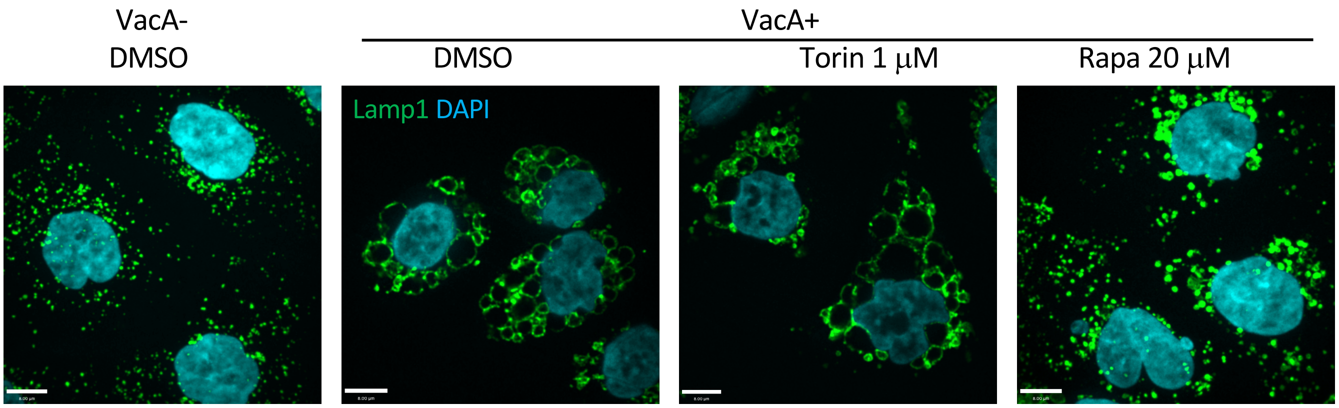

Supplementary Figure 2: Lamp1 staining of AGS cells after 4h VacA- or VacA+ incubation followed by 3h of DMSO, Torin (1 mM) or rapamycin (Rapa, 20 mM) treatment.

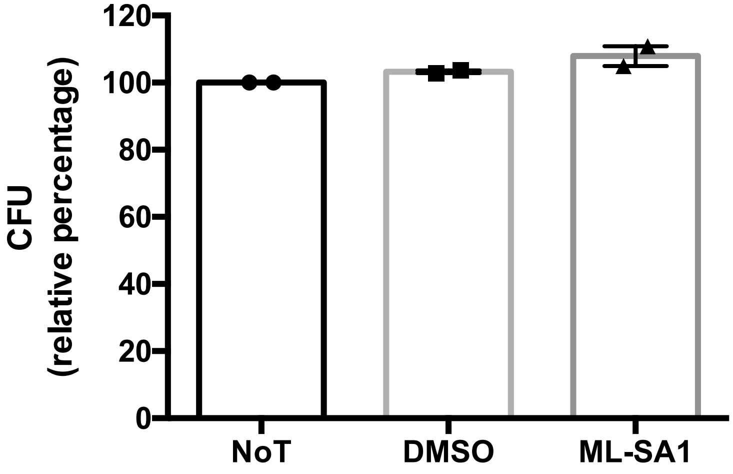

Supplementary Figure 3: Quantification of *Hp* growth after 4 h incubation with DMSO or ML-SA1 (20 mM). Graph shows the relative percentage of CFU compared with a control culture (NoT = no treatment) considered as 100%.

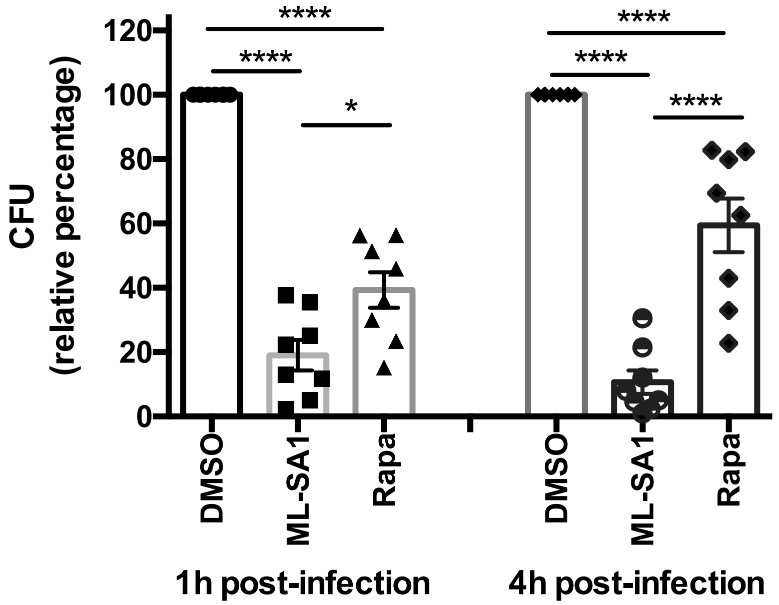

Supplementary Figure 4: HeLa cells infected with *Lm* were incubated with gentamycin and treated with ML-SA1 (20 uM), rapamycin (Rapa, 100 nM) or vehicle control (DMSO). Treatments were added 1h or 4 h post-infection and maintained for a total of 24 h infection. Intracellular bacteria were retrieved and CFU quantified. Graph shows relative percentage of CFU (mean +/- SEM of 8 independent experiments) considering 100 the bacteria retrieved from DMSO-treated cells.

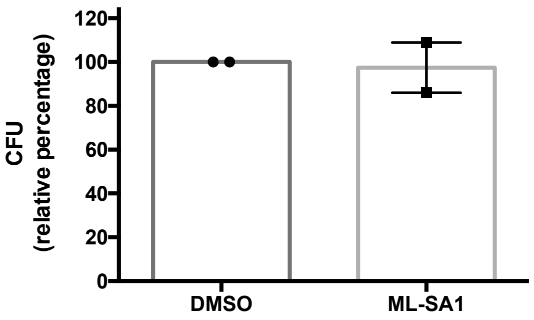

Supplementary Figure 5: HeLa cells infected with *St* were incubated with gentamycin. ML-SA1 (20 mM), or vehicle control (DMSO) were added 1h post infection and maintained for a total 5h infection. Intracellular bacteria were retrieved and CFU quantified. Graph shows relative percentage of CFU considering 100 the bacteria retrieved from DMSO-treated cells.

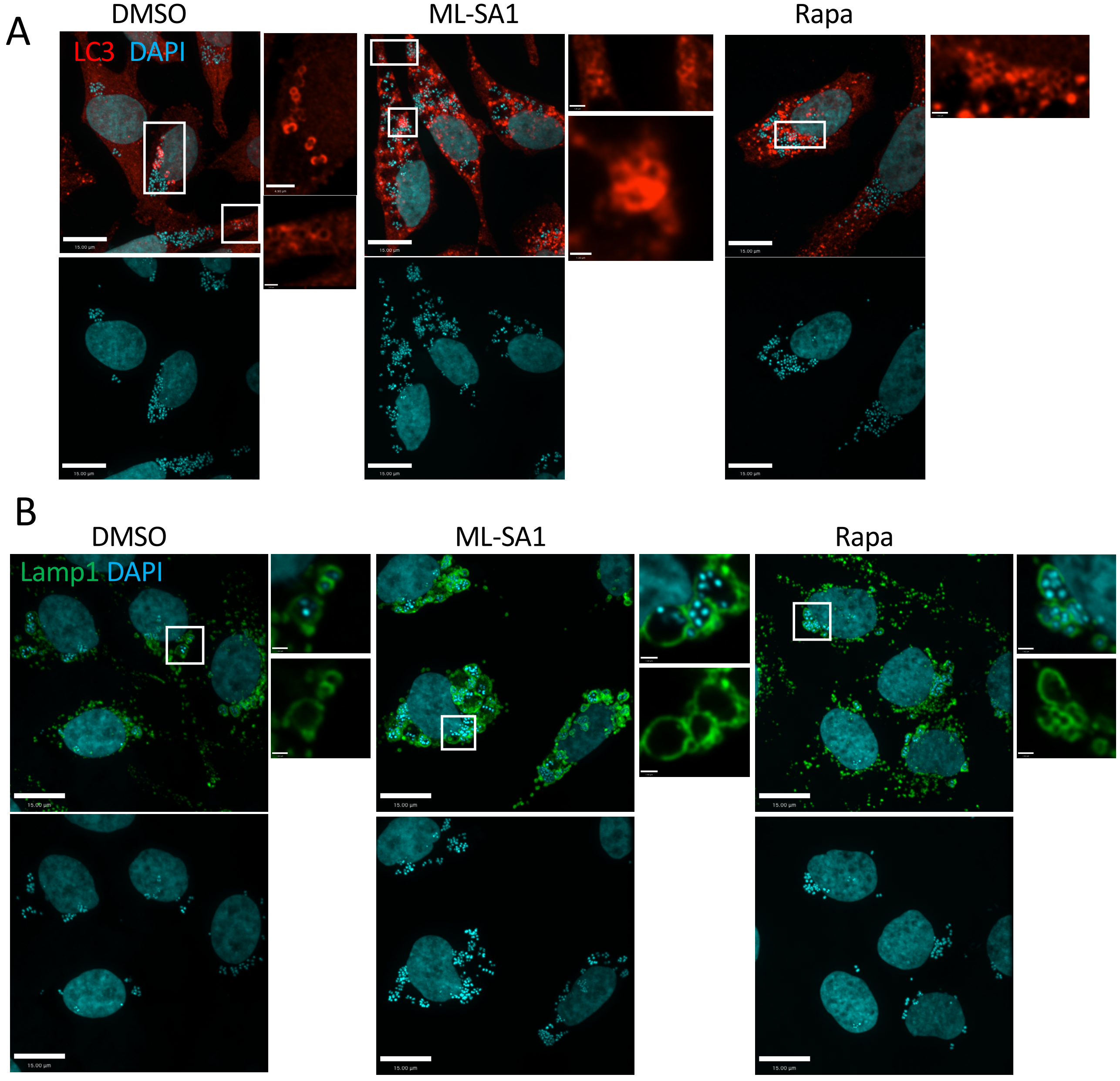

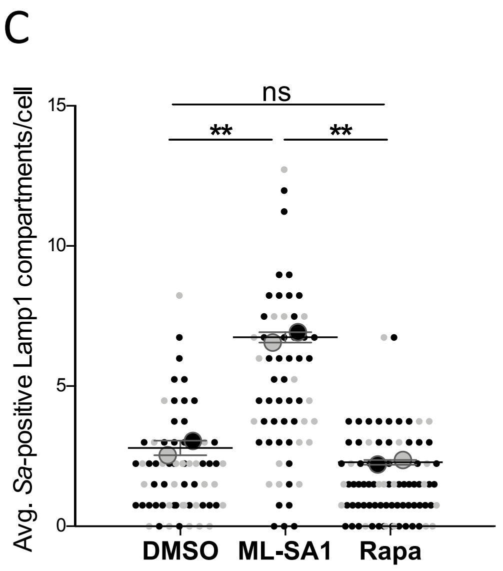

Supplementary Figure 6: HeLa cells infected with *Sa* were incubated with gentamycin and treated for 20h with ML-SA1 (20 mM), rapamycin (Rapa, 100 nM) or vehicle control (DMSO). (**A**) LC3 and DAPI staining. Images of DAPI channel to visualize bacteria are included at the bottom. Higher magnification of the selected areas are shown on the right of each panel. (**B**) Lamp1 and DAPI staining. Images of DAPI channel to visualize bacteria are included at the bottom. Higher magnification of the selected areas are shown on the right of each panel. (**C**) *Sa*-positive Lamp1 compartments/cell quantification in *Sa*-infected HeLa cells treated as (B) (n=2, each color corresponds to 1 experiment, big symbol representing the mean and small symbols the scatter dot plot. Lines show the mean +/- SEM).
