# Supplementary figures and images for: Survival of intracellular pathogens in response to mTORC1- or TRPML1-TFEB-induced xenophagy
Source: Autophagy Rep. 2023 Mar 19;2(1):2191918. doi: 10.1080/27694127.2023.2191918 (PMC12039413; doi:10.1080/27694127.2023.2191918)

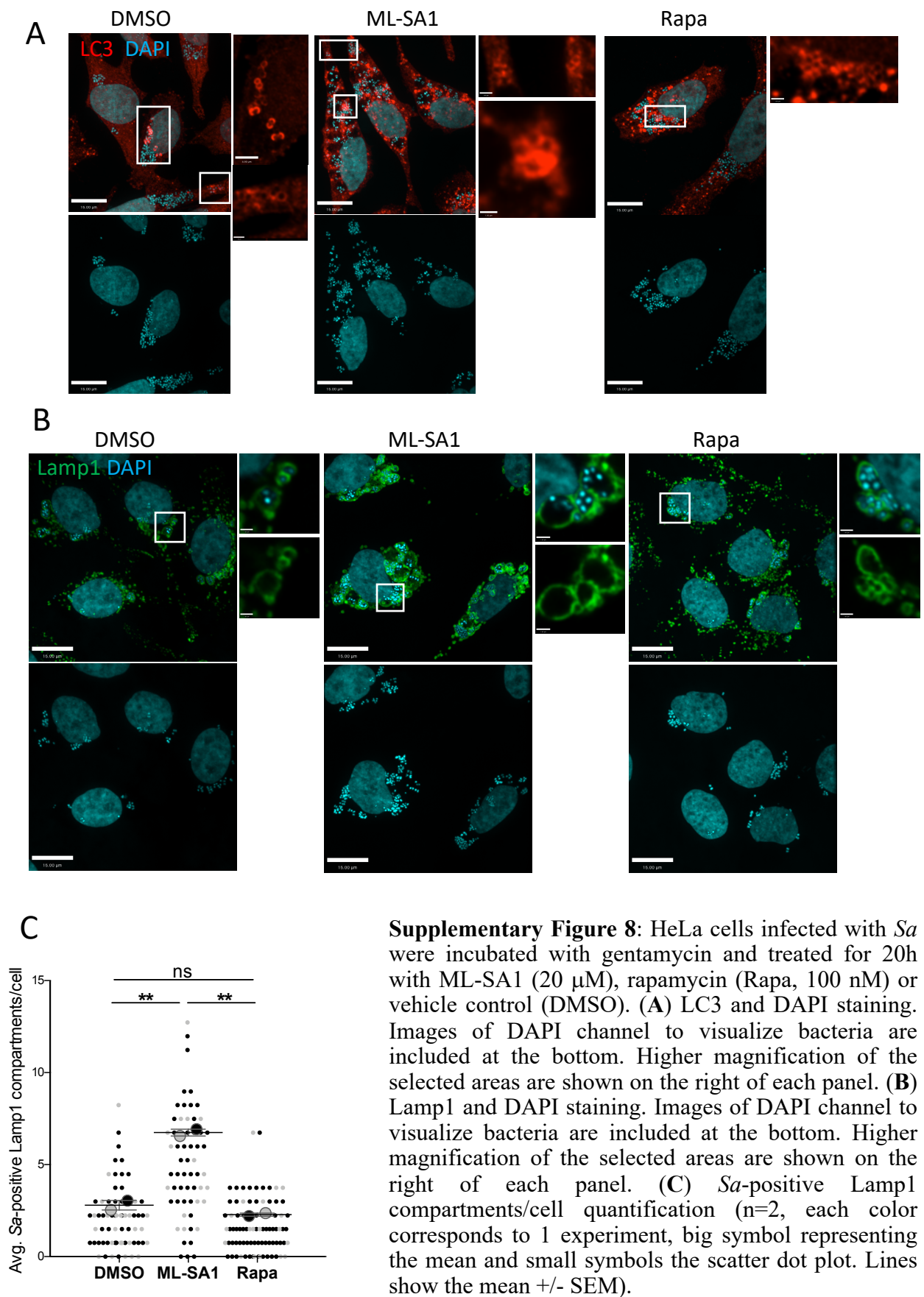

Supplement: Supplemental Material [file KAUO_A_2191918_SM6084.zip › Revised FigS8.pdf]
